# Transcriptome sequencing assisted discovery and computational analysis of novel SNPs associated with flowering in *Raphanus sativus* in-bred lines for marker-assisted backcross breeding

**DOI:** 10.1038/s41438-019-0200-0

**Published:** 2019-11-01

**Authors:** Jinhee Kim, Abinaya Manivannan, Do-Sun Kim, Eun-Su Lee, Hye-Eun Lee

**Affiliations:** Vegetable Research Division, National Institute of Horticultural and Herbal Science, Rural Development Administration, Jeonju, 55365 Republic of Korea

**Keywords:** Flowering, Plant breeding

## Abstract

The sequencing of radish genome aids in the better understanding and tailoring of traits associated with economic importance. In order to accelerate the genomics assisted breeding and genetic selection, transcriptomes of 33 radish inbred lines with diverse traits were sequenced for the development of single nucleotide polymorphic (SNP) markers. The sequence reads ranged from 2,560,543,741 bp to 20,039,688,139 bp with the GC (%) of 47.80–49.34 and phred quality score (Q30) of 96.47–97.54%. A total of 4951 polymorphic SNPs were identified among the accessions after stringent filtering and 298 SNPs with efficient marker assisted backcross breeding (MAB) markers were generated from the polymorphic SNPs. Further, functional annotations of SNPs revealed the effects and importance of the SNPs identified in the flowering process. The SNPs were predominantly associated with the four major flowering related transcription factors such as MYB, MADS box (AG), AP2/EREB, and bHLH. In addition, SNPs in the vital flowering integrator gene (FT) and floral repressors (EMBRYONIC FLOWER 1, 2, and FRIGIDA) were identified among the radish inbred lines. Further, 50 SNPs were randomly selected from 298 SNPs and validated using Kompetitive Allele Specific PCR genotyping system (KASP) in 102 radish inbred lines. The homozygosity of the inbred lines varied from 56 to 96% and the phylogenetic analysis resulted in the clustering of inbred lines into three subgroups. Taken together, the SNP markers identified in the present study can be utilized for the discrimination, seed purity test, and adjusting parental combinations for breeding in radish.

## Introduction

Radish (*Raphanus sativus* L., 2*n* = 18) is an important root vegetable in the Brassicaceae family. The tap root of radish has been widely consumed dietary part worldwide. Similarly, radish leaves, seedlings, siliques, and sprout also possess dietary and medicinal importance^1^. Due to the higher consumption rate, several breeding efforts were implemented as well as researched to introduce cultivars with diverse phenotypic and economic qualities. Especially, the qualities such as shape, size, color, flowering period, anthocyanin contents of roots, and higher secondary metabolites have become the interest of plant breeders^2^. Researches on the construction of linkage maps using various molecular markers such as simple sequence repeats (SSR), amplified fragment length polymorphism (AFLP), and single nucleotide polymorphism (SNPs) were conducted to boost up the molecular breeding of radish. Further, QTL maps were constructed for the analysis of disease resistance, pathogen resistance, and cadmium accumulation in radish^3–6^. In recent days, NGS approaches like transcriptome sequencing resulted in the generation of comprehensive sequence data in several horticultural plants such as pepper, cucumber, and eggplant,^7–9^. In addition, the transcriptomics information acts as vital resources for the development of molecular markers in numerous plant species^3,10,11^. Among the molecular markers, SNP is widely employed in the molecular breeding programs now a days due to its abundance, biallelic nature, and low mutation rate^12^. Moreover, the amenability of SNP markers for high-throughput assays makes it as a marker of choice for plant breeders. In addition, the SNP genotyping is easily applicable for large populations or germplasm collection especially for the construction of genetic maps, genome wide association studies, and marker assisted breeding in cost effective manner^13^. In radish, the SNP markers were utilized for the construction of expressed sequence tags based genetic maps which aided in the genomic comparison among Brassica species^14^.

Flowering is a vital and complex process in plant propagation controlled by diverse internal gene regulatory networks and environmental cues. For instances in *Arabidopsis thaliana*, flowering is regulated by approximately 174 genes involved in six vital pathways such as photoperiod, vernalization, age, autonomous, gibberellin, and ambient temperature related pathways^15^. In Brassicaceae plants like radish, the progression from bolting to blooming determines the conversion from the vegetative to reproductive phase^16^. Therefore, the molecular rationale behind the flowering enables the breeders to efficiently increase the quality and yield by maintaining the stability between the vegetative and reproductive phases. The flowering habit of radish particularly with respect to flowering time and variable responses to photoperiod, and temperature has been in similar manner with the closely related plants such as *Arabidopsis thaliana*, *Brassica oleracea*, and *Brassica rapa*^16^. Recently, the availability of radish genome sequence promotes the understanding of molecular mechanisms associated with the regulation of flowering genes. According to Jeong et al.^17^, the radish genome consisted of 290 flowering related genes. However, little information is available on the molecular rationale behind the flowering regulation in radish and the synergistic involvement of genes associated with flowering related pathways. Especially, the knowledge on molecular markers associated with the flowering genes and transcription factors in radish is scarce. Therefore the present study has aimed to investigate the SNPs related to flowering in early, middle, and late flowering accessions of radish inbred lines.

Several approaches were used to authenticate the SNP in a population. However, the selection of genotyping technique depends on the size of the sample, number of markers, assay platform, cost effectiveness, and accuracy^18^. Kompetitive allele specific PCR (KASP) assay employs the advantages of both polymerase chain reaction and fluorescent SNP genotyping approach. The KASP approach is a flexible SNP genotyping system that can be performed on various set of markers^19^. Overall, the objective of the present study is to discover SNP markers from 33 inbred lines of radish by utilizing the information obtained from the transcriptome sequencing, functional annotation of the SNPs, and investigation of SNPs associated with flowering. Further, the SNP marker data were used for designing the probes for marker assisted backcrossing (MAB) and genotyped using KASPar assay. The outcomes of the current endeavor will benefit the radish breeding and screening of cultivars with high economic importance.

## Results

### Transcriptome analysis and SNP discovery of *R. sativus*

In the current study 33 *R. sativus* lines were selected based on the economically important phenotypic traits such as skin color (blank, red, yellow, green, and white, green-white, purple), leaf shape, root shape and length, root flesh color, maturity, and flowering pattern (Supplementary Table 1). The transcriptome sequencing produced the maximum trimmed reads of 20,039,688,139 bp to a minimum of 2,560,543,741 bp among the radish accessions (Supplementary Table 2). Moreover, the GC (%) of the sequences ranged from 47.80 to 49.34. The phred quality scores denoted the high quality sequencing results obtained with the values ranging from 98.99–99.32% for Q20 and Q30 was observed to be 96.47–97.54%. After trimming process, the reads were mapped to the radish reference sequence (Supplementary Table 3). Overall mean of processed and mapped reads were 28,683,155 and 23,825,281 respectively with the multiple reads ranging from 1,450,482 to 10,408,340. The average mapping ratio of the 33 accessions with the reference genome is 80.89%. The SNPs were discovered using the Samtools and the in-house pipelines. Further, the identified crude SNPs were filtered based on the criteria such as homozygous and diallelic SNPs, SNPs with the ability to classify at least 15 accessions, segregation ratio, flanking SNPs, and SNPs suitable for marker assisted backcross breeding. The number of filtered SNPs along with the filtering percentage has been listed in Table 1. Initially, 901522 SNPs have been screened as homozygous and diallelic SNPs and a total of 802404 SNPs contained the ability to distinguish at least 15 different accessions. Subsequently, 67568 SNPs passed the third filter (segregation ratio) and the adjacent SNP markers within the window length of 60 bp were eliminated in order to achieve the highly reliable SNP marker set with enhanced genotyping efficiency which remained the 4951 potential SNPs.

**Table 1:** List of SNPs filtered in the radish inbred lines

Filtering criteria	No. of SNPs favored the filtering criteria	No. of filtered SNPs	Filtering percentage (%)
Genotype/Allele	901522	255861	22.1
Distinguishable species	802404	99118	11.0
Segregation ratio	67568	734836	91.6
Flanking SNP	4951	62617	92.7
QC/MAB suitable SNP	298	4653	94.0

**Fig. 1:**
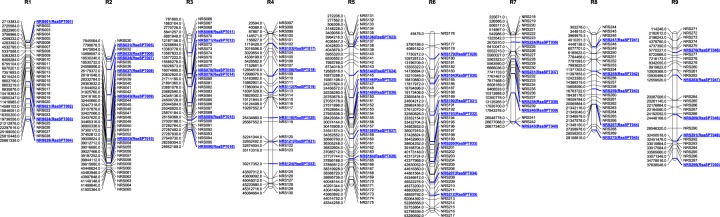
Physical map of radish with the SNP markers located across the chromosome. Blue colored SNP locus denotes the markers used for KASP validation

Finally, for the selection of MAB marker set 298 suitable SNP markers evenly distributed across all the nine chromosomes have been filtered (Fig. 1, Supplementary Table 4). The number of SNPs varied among the chromosomes with the densities of SNPs ranging between 0.74 and 1.12. Among the chromosomes, the highest density of SNPs (1.12) were identified in chromosome 1 and chromosome 4 displayed the lowest SNP density (0.74) (Table 2). The physical map of radish along with the positions of 298 SNP markers selected for MAB probe designing were shown in Fig. 1.

**Table 2:** Chromosome-wise distribution of SNPs in *R. sativus*

Chromosome	SNPs	Size	SNP density	KASP markers
Chr.1	29	25.86	1.12	4
Chr.2	36	41.50	0.87	6
Chr.3	31	28.95	1.07	6
Chr.4	34	46.08	0.74	6
Chr.5	45	45.34	0.99	6
Chr.6	42	53.29	0.79	7
Chr.7	26	26.68	0.97	5
Chr.8	27	29.11	0.93	5
Chr.9	28	37.64	0.74	5
Total	298	334.46	0.89	50

### Functional annotation of SNPs

**Fig. 2:**
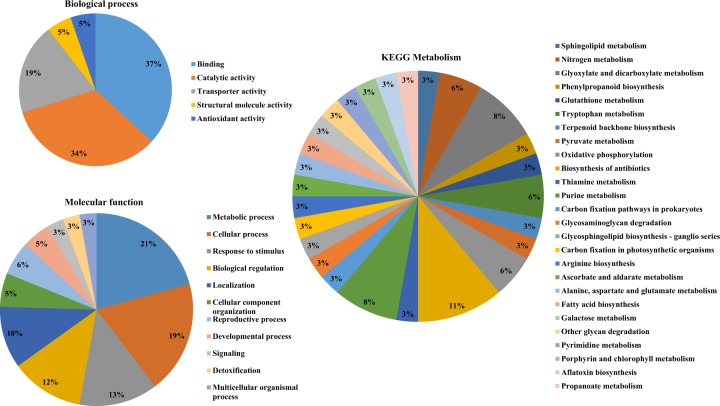
Functional annotation of SNPs. The Gene ontology analysis results illustrating the involvement of SNPs in vital biological process (**a**), molecular functions (**b**) and KEGG metabolisms (**c**)

The functional annotation of 4951 filtered SNPs were achieved by extracting the scaffolds containing the SNPs using the Bedtools and used as the query for initial sequence similarity searches and gene ontology annotations. The SNPs have been involved in important biological processes with a major numbers in metabolic process (21%), cellular process (19%), response to stimulus (13%), and biological regulations (12%), etc. (Fig. 2). Likewise, the SNPs were associated with the vital molecular functions like binding (37%), catalytic activity (34%), transporter activity (19%), structural molecule activity and antioxidant activity (5%). Further, the KEGG pathway analysis of the SNPs illustrated the association with diverse metabolic pathways such as biosynthesis of antibiotics, glyoxylate and dicarboxylate metabolism, purine metabolism, nitrogen metabolism, tryptophan metabolism, oxidative phosphorylation, sphingolipid metabolism, phenylpropanoid biosynthesis, phenylpropanoid biosynthesis, glutathione metabolism, terpenoid backbone biosynthesis, pyruvate metabolism, thiamine metabolism, carbon fixation pathways, glycosaminoglycan degradation, amino acid biosynthesis, fatty acid biosynthesis, galactose metabolism, pyrimidine metabolism, porphyrin and chlorophyll metabolism, aflatoxin biosynthesis, and propanoate metabolism. Hence, the functional gene ontology study aided in the global view of the importance of the filtered SNPs in the radish inbred lines.

### Impact analysis of SNPs using SnpEff

**Fig. 3:**
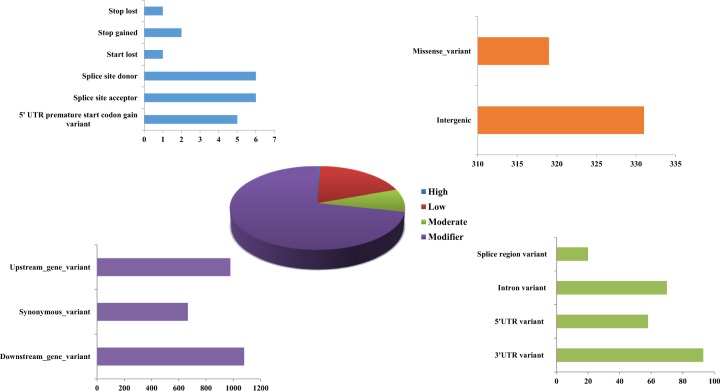
Impact of SNPs in the functionality of the genes predicted using the SnpEff tool

Based on the SnpEff results, the overall impact of the SNPs on the functionality of the genes were categorized into modifier (71.1%), low (19.01%), moderate (8.84%), and high (0.44%) impacts (Fig. 3). The majority of SNPs with modifier effect were observed as downstream gene variants (29.7%), upstream gene variants (27.0%), and synonymous variants (18.3%). The low effect SNPs mostly occurred as intergenic variants (9.1%) and missense variants (8.8% %) whereas the moderate impact SNPs were observed as UTR variants (4.1%), intron (1.9%), and splice region variants (0.05%). Moreover, the high impact SNPs were identified as 5′ UTR premature start codon gain variant (0.13%), splice site acceptor (0.16%), splice site donor (0.16%), start lost (0.027%), stop gained (0.55%), stop lost variants (0.027%).

### SNPs associated with flowering related transcription factors in radish inbred lines

**Fig. 4:**
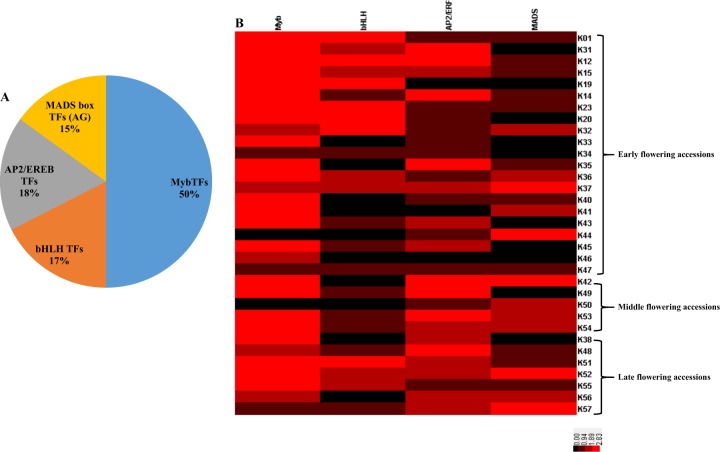
The SNPs associated with the flowering related transcription factors. The percentage of TFs related SNPs associated with flowering (**a**) and the heat map illustrating the individual TFs related SNPs associated with flowering identified in the early, middle, and late flowering accessions of radish inbred lines (**b**)

The identified SNPs were mostly associated with the four major transcription factors such as MYB, MADS box AGAMOUS (AG), APETELA2/ethylene responsive element binding (AP2/EREB), and basic Helix-Loop-Helix (bHLH) (Fig. 4). These transcription factors regulate various physiological processes including flowering responses. In radish, the identified transcription factors played vital role in the flowering and bolting. The SNPs associated with MYB transcription factors were observed in high percent (50%) followed by AP2/EREB (18%), bHLH (17%), and MADS box (AG) (15%). The SNPs related to MYB transcription factors were identified in the majority of the accessions except K44 (early flowering) and K50 (middle flowering). The SNPs associated with AP2/EREB transcription factors were present in all the middle and late flowering accessions apart from three early flowering accessions such as K19, K41, and K46. Moreover, the bHLH transcription factors related SNPs were absent in six early flowering accessions (K33, K35, K40, K41, K44, K46), two middle flowering accessions (K42 and K50), and two late flowering accessions (K38 and K56) respectively. On comparison with the other transcription factors, MADS box transcription factors were less observed in early flowering accessions (nine accessions lacked the SNPs associated with MADS box transcription factor). However, the MADS box TFs were observed in the middle and late flowering accessions except K49 (middle flowering accession) and K38 (late flowering accessions).

### SNPs associated with floral signal repressors and integrators genes in radish inbred lines

**Fig. 5:**
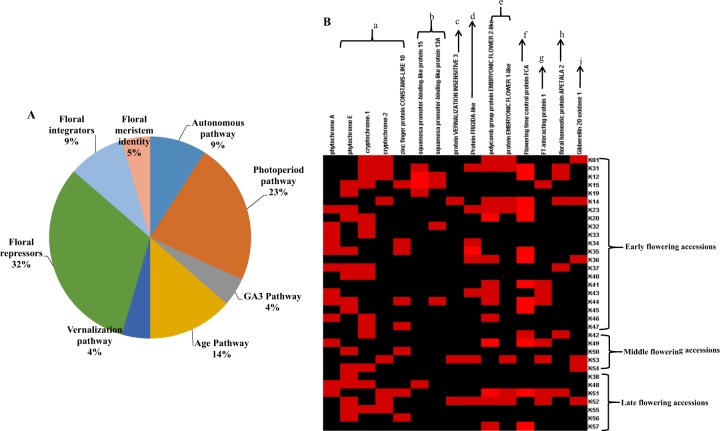
The SNPs associated with the flowering genes. The overview of flowering associated SNPs related to different flowering pathways (**a**). SNPs identified in flowering related genes, floral integrators, and repressors involved in the vital flowering pathways are represented as a, photoperiod pathway; b, age pathway; c, vernalization pathway; d and e, floral repressors; f, autonomous pathway; g, floral integrators; h, floral meristem identity; i, gibberellin pathway in the early, middle, and late flowering accessions of radish inbred lines (**b**)

Similarly SNPs related to flowering genes involved in the major flowering pathways were identified in all the accessions of radish employed in this study. In detail, SNPs in the genes involved in the flowering pathways such as photoperiod pathway, autonomous pathway, GA_3_ pathway, vernalization pathway, and age pathway were identified. In addition, the SNPs were also identified in the vital floral integrators, repressors, and floral meristem identity genes in radish (Fig. 5). A higher number of SNPs were associated with the floral repressors (32%) followed by the photoperiod pathway genes (23%) and lesser number of SNPs were associated with the GA_3_ and vernalization pathways (4%) were identified. Among the radish inbred lines, K31 (early flowering accession) consisted of majority of SNPs associated with flowering genes whereas K38 (late flowering accession) possessed the least SNPs related to flowering. The SNPs linked to the floral repressors such as polycomb EMBRYONIC FLOWER 1, 2, and FIRGIDA like protein were identified in the early, middle, and late flowering accessions. Among the floral repressors, the majority of the SNPs were associated with polycomb EMBRYONIC FLOWER 2 and 1 followed by FRIGIDA like protein. Moreover, the SNPs related to polycomb EMBRYONIC FLOWER 2 were observed in more number than the polycomb EMBRYONIC FLOWER 1 among the early, middle, and late flowering accessions. On the other hand, six early flowering accessions possessed SNPs linked to FRIGIDA however only one was observed in the middle flowering accession (K53) and none in the late flowering accessions. The floral integrator namely FT interacting protein 1 related SNPs have been identified in four early flowering accessions (K12, K40, K41, and K43), two middle flowering accessions (K48 and K51), and two late flowering accessions (K48 and K51).

### SNPs associated with flowering related pathways in radish inbred lines

**Fig. 6:**
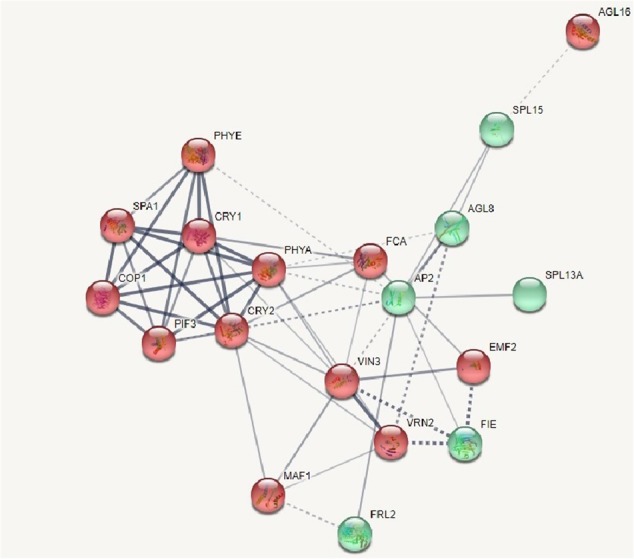
The interaction analysis of flowering related proteins and transcription factors identified in the radish inbred lines clustered into two groups. The red spheres denote cluster 1 and green spheres represents cluster 2. The strong weighted lines represents strong interaction between the proteins and the dashed lines denotes the weaker interactions. The clusters have been generated using Kmean clustering algorithm from STRING database

A wide range of SNPs associated with photoperiod pathway of flowering genes such as phytochrome A (PHY A), phytochrome E (PHY E), cryptochrome 1 (CRY 1), and cryptochrome 2 (CRY 2) have been identified in the radish inbred lines. However, three accessions such as K36 (early flowering), K41 (early flowering), and K57 (late flowering) lacked the SNPs related to photoperiod pathway of flowering. On the other hand, the accession K38 (late flowering) consisted of only one SNP related to flowering genes which corresponded to PHY E. In addition, age pathway of flowering related genes such as squamosa promoter binding like protein 13A and 15 were identified with SNPs. However, SNPs related to both squamosa promoter binding like protein13A and 15 were noted in the early accessions K12 and K15 whereas K31, K19, K32, and K44 encompassed SNP related to squamosa promoter binding like protein 15. On the contrary, the SNPs linked to squamosa promoter binding like proteins were absent in the middle flowering accession and K48 (late flowering) possessed one squamosa promoter binding like protein 15 related SNP. The SNPs associated with vernalization insensitive 3 gene involved in the vernalization mediated flowering pathway were identified in three radish accessions such as K14 (early flowering), K53 (middle flowering), and K52 (late flowering). Moreover, SNPs related to gibberellin 20 oxidase 1 a vital enzyme in GA_3_ flowering pathway were observed in three early flowering accessions (K1, K14, and K36), two middle flowering accessions (K53 and K54), and one late flowering accession (K52). In addition SNPs related to flowering time control protein FCA involved in the autonomous pathway of flowering has been identified in 12 early flowering accessions, two middle flowering accessions, and one late flowering accession of radish. Moreover, the interaction analysis of proteins associated with flowering related pathways have been classified into two clusters using K-mean clustering algorithm based on the distance matrix resulted from the STRING scores. The algorithm interprets the scores and clusters the interacting proteins with higher global score into same group. In the present study, the first group (red color spheres) consisted of vital proteins involved in the photoperiod pathway, vernalization pathway, and floral integrator, etc. The second group (green spheres) encompassed the proteins involved in the age pathway, floral meristem identity, and others (Fig. 6). The network displayed the complex association and strong interaction between the proteins related with the flowering pathways.

### Validation of SNPs using KASP assay

**Fig. 7:**
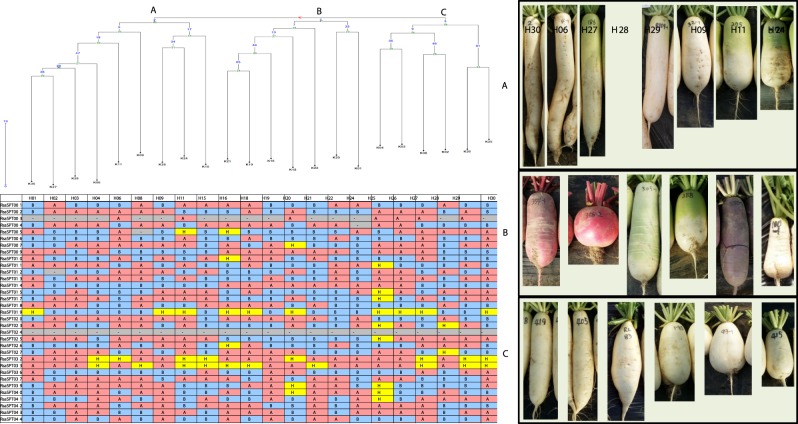
SNP genotyping using KASP analysis and phylogenetic tree of radish inbred lines. The genotype data and phylogenetic tree of the inbred lines along with the phenotype. Only a portion of the results are shown due to the crude data size

To validate the SNPs, 50 SNPs were randomly selected from the 298 SNPs to develop Kompetitive Allele Specific PCR genotyping system (KASP) markers (Supplementary Table 5). Four to seven SNPs were selected from each chromosome for the analysis. The genomic DNA of 102 radish inbred lines obtained from several seed companies were used in this experiment (data not shown). The homozygosity of the inbred lines varied from 56 to 96%. In order to determine whether the genotyping data of the 50 KASP markers could influence the phenotype of radish accessions, phylogenetic analysis was performed. For the phylogenetic analysis, 22 radish inbred lines were employed. According to the genotyping result, three subgroups (A, B, and C) were identified using the KASP markers (Fig. 7). Five radish lines with long root length were grouped in A. In this group, five among eight inbred lines displayed green to pale green color near the crown. These lines have largely favored phenotype for Kimchi production in Korea. Interestingly, red, purple, and green colored skin radish was clustered in group B. The rest of the lines with pure white colored skin and flesh were grouped in C. Four out of six were round shaped radish in group C. Further linkage analysis is needed to develop trait based SNP markers. In addition, the 50 SNP markers can be utilized in radish cultivar discrimination, seed purity test, and adjusting parent combination for breeding.

## Discussion

High throughput transcriptome sequencing using Illumina technology aids in the generation of large scale transcript sequence information for molecular marker development. The Illumina based transcriptome sequencing has been employed in several economically important horticultural crops such as *Raphanus sativus*^11^, *Brassica napus*^20^, *Brassica oleracea*^21^, *Capsicum aunnum*^22^, and *Solanum lycopersicum*^23^. Owing to the experimental, algorithmic advancements, and cost-effectiveness benefits the implementation of Illumina approach in sequencing model and non-model organisms^3^. In the current study, the transcriptome of 33 radish inbred lines native to Korea, Japan, and China with diverse traits such as flowering time (early, middle, and late), growth rate (fast and slow), skin color (white, green, and purple), flesh color (white, green, and purple), root length (short, middle, and long), leaf shape (lyrate and undivided) were sequenced.

Discovery of SNPs from the transcriptome sequence data yielded a total of 901522 crude SNPs which upon stringent filtering reduced to 4951 polymorphic SNPs across the inbred lines (Supplementary data 1). Subsequently, from the filtered 4951 SNPs, 298 SNPs distributed evenly across the length of the chromosome were selected for the generation of marker assisted backcross marker probes. Even though, the markers are evenly distributed the number of SNPs varied among the chromosome with the higher number in chromosome 1 and lesser in chromosome 4. Similar observations have been reported by Kim et al.^24^, in Chinese cabbage. Molecular markers are vital tools for the investigation of genetic diversity, construction of linkage maps, association and evolutionary studies^25^. Among the molecular markers, SNPs are considered as marker of choice because of its numerous benefits such as automation for high throughput assays, diallelic nature, and accuracy^26^. Recently, Wang et al.^3^, have developed SNP markers from transcriptome sequences for the germplasm identification in radish. However, in the present study the SNPs have been functionally annotated for obtaining the comprehensive view of the genes associated with the SNPs. The functional annotation of the filtered SNPs revealed the association of SNPs with genes involved in numerous vital biological processes and molecular functions (Supplementary data 2 and supplementary data 3).

Among the molecular functions annotated, majority of SNPs were linked to genes involved in binding function such as the binding of nucleotide, nucleoside, metal ions, organic cyclic compounds, heterocyclic compounds, proteins, lipids, carbohydrates, co-factors, and co-enzymes (Supplementary Fig. 1). Similarly, the functional annotations of SNP markers were reported in Korean inbred onions^27^ and capsicum^18^. Moreover, the availability of radish genome sequence aided in the homology search and assignment of various GO terms to the SNP-associated scaffolds. In addition, the KEGG pathway analysis illustrated the involvement of SNPs in vital metabolic pathways. Hence the functional annotations of SNPs could render a valuable resource to understand the importance of SNPs in the radish inbred lines. Moreover, the impact analysis of SNPs using SnpEff tool revealed the effects of SNPs on the functionality of the genes. The majority of SNPs discovered in the present study were classified with modifier effects. According to Bhardwaj et al.^28^, the impacts of SNPs can be distinguished as high (directly affect the gene function with high level of functional consequences), low (minimal impact on the function of gene or unaltered function), modifier (tends to modify the functionality of the genes) wherein in the present study the SNPs with modifier effects were identified predominantly as the upstream and downstream variants.

The radish inbred lines consisted of varied flowering time characteristics such as early, middle and late flowering. The number and occurrence of SNPs associated with the flowering related transcription factors and genes varied significantly among the accessions. Among the 33 inbred lines 21 accessions belonged to early flowering, five middle flowering accessions, and seven late flowering accessions respectively. Flowering time is crucial for the radish breeding which determines the quality and yield, therefore the SNPs associated with flowering have been investigated. The molecular regulation of flowering is a complex processes which involves the internal and external environmental cues such as ambient temperature, light, and photoperiod. Several studies have been attempted to elucidate the flowering related genes and molecular regulatory networks that control flowering time^16,29,30^. Particularly in *Arabidopsis thaliana* the flowering enabling and promoting pathways which includes six major pathways such as photoperiod, age, autonomous, vernalization, gibberellin pathway, and meristem identity along with their crosstalk have been investigated in detail ^31^.

The signals associated with flowering from the abovementioned pathways are integrated and regulated by floral integrators such as FLOWERING LOCUS T (FT), LEAFY (LFY), and SUPPRESSOR OF OVEREXPRESSION OF CO1 (SOC1)^31^. In the current study the SNPs were identified in the FT, which is the central node of the floral integrators. Moreover, SNPs associated with the floral activator CONSTANS (CO)-like 10 were identified in radish. The CO acts as the positive regulator of FT and negative regulator of the floral repressor FLOWERING LOCUS (FLC)^31^. In addition, the CO is considered to link the plant circadian rhythm and flowering control. Moreover, the florigene FT perceives and converges the photoperiodic signals and transport to the shoot apex for the initiation of flowering^15^. However in radish inbred lines SNPs in FRIGIDA were observed along the accessions. In general, the flowering is delayed by the up regulation of the FLC which is activated by the FRIGIDA^31^. Other than FRIGIDA the SNPs related to EMBRYONIC FLOWER 1, 3 which repress flowering have been discovered in the radish inbred lines.

Moreover, the vernalization pathway suppresses the FLC locus by the vernalization insensitive 3 (VIN3) mediated histone modification and promotes flowering^15^. In the current study SNPs associated with VIN3 in radish inbred lines were observed. Likewise, the photoperiod pathway controls flowering in Arabidopsis by channeling a series of signaling cascade mediated by GIGANTEA (GI) and CO^31^. In addition, the gibberellin 20 oxidase, a rate limiting enzyme that catalyzes numerous reactions in the biosynthesis of gibberellins by oxidizing its precursors^15^. In general, the onsite of floral induction consists of higher levels of bioactive gibberellins which aids in the floral initiation. Hence, the mutation in the gibberellin 20 oxidase could influence the flux of gibberellins in the early, middle, and late flowering accessions of radish inbred lines. The squamosa promoter binding protein like (SPL) involved in the aging pathway promotes flowering by increasing the expression of LFY, FRUITFUL (FUL), and SOC1^15^. Apart from the abovementioned pathways, the meristem response also significantly influence flowering. For instance, the induction of flowering requires the transition of shoot apical meristem from vegetative to inflorescence meristem to bear the flowers. This transition requires the higher expression of genes related to meristem response like APETELA 2^15^. Moreover, the meristem response genes are also involved in the complex interaction of transcription factors such as AG, SPL, and LFY^32^. In addition differences in the SNPs associated with flowering related transcription factors (AG, AP2/EREB, and bHLH) were identified among the early, middle, and late flowering accessions of radish. The importance of transcription factors related to flowering is inevitable for the regulation of flowering related genes in radish^33^. The occurrence of SNPs related to flowering in the radish inbred lines could be utilized for the construction of QTL maps and screening of radish cultivars for the late flowering and bolting varieties with commercial importance.

Moreover the platform for the genotyping of SNPs relies on various factors such as precision, reproducibility, and multiplexing, high throughput, time and cost effectiveness^19^. The utilization of cost effective KASP assay demonstrated the relationship among the collection of radish inbred lines. Similarly, the SNP based KASP analysis rendered the selection of chickpea^19^, maize^34^, rice^35^, and tomato^36^ for the diversity analysis, genetic purity test, and quality control to enhance the molecular breeding. Further, the genetic relationship among the radish inbred lines using KASP assay classified the radish lines into three groups. The group A consisted of radish accessions with long root, skin color varying from green to pale green color which is largely used for the Kimchi production in Korea. Moreover, the second group consisted of red, purple, and green skinned radish. The third group encompassed radish lines with pure white skin and round shaped. Similarly, Cheon et al.^37^, utilized the SNP based KASP markers for the phylogenetic analysis of Japonica rice varieties. According to Kim et al.^26^, the SNP markers derived from the transcriptome sequence of Chinese cabbage accession facilitated the understanding of genetic relationship among the accessions. In addition, the outcomes of the present study can promote the cultivar discrimination, seed purity test, and adjusting parent combination for radish breeding.

## Materials and methods

### Plant materials and RNA isolation

*Raphanus sativus* inbred lines were selected based on the economically important traits such as skin color, leaf shape, root shape and length, root flesh color, maturity, and flowering pattern. The flowering pattern varied from early (flowers after 100–160 days of sowing), middle (flowers after ~180 days of sowing), and late flowering (flowers after more than 200 days of sowing) accessions. The plant materials were provided by the Hankook Seed. Co., Ltd. and Neoseed Co., Ltd. (Ansung, Republic of Korea) and all the plants were maintained under natural conditions. The inbred lines were produced after seven to 11 generations of selfing. For total RNA isolation, young leaves from 14 days old plants were collected and immediately homogenized using liquid nitrogen. Further, the RNA was extracted using GeneAll Hybrid-RTM kit (GeneAll Biotechnology Co., Ltd., Daejeon, Korea) according to the manufacturer’s protocol. The quality and quantity of the extracted RNA has been determined using the Nano Drop analyzer (NanoDrop Technologies, Wilmington, DE, USA) and employed for transcriptome sequencing.

### Transcriptome sequencing

The Illumina Genomic mRNA shotgun library process was performed at the National Instrumentation Center for Environmental Management (Seoul National University, Seoul, Korea). An Agilent 2100 Bioanalyzer (Agilent Technologies, Palo Alto, CA, USA) was used for quality control of the RNA. We selected high-quality RNA samples showing RIN (RNA integrity number) values over 7 and 28S/18S rRNA ratios over 1. cDNA synthesis and RNA-seq library construction were performed at the National Instrumentation Center for Environmental Management based on the protocols of the Illumina HiSeq2500 (Illumina, San Diego, CA, USA). Sequencing was performed by 101 bp paired-end (PE) sequencing. The DNA fragment size was 300 to 600 bp. The sequencing data from three lanes were merged for further analysis.

### Data preprocessing and SNP discovery

The raw data generated from the Illumina Hiseq2500 was trimmed to remove low quality data using Trimmomatic 0.32^38^. Initially, the adapter sequences were removed followed by the read end correction and trimming. The sliding window trimming approach with the window length of 4, mean quality of 15, and minimum length of 36 bp were employed for obtaining the clean trimmed reads. Data below a phred score of 20 was eliminated. Subsequently, the trimmed reads were mapped to the radish reference sequence retrieved from Radish Genome Database at http://www.radish-genome.org. TopHat 2.0.13^39^ and BWA 0.7.8-r455 (Burrows-Wheeler Aligner) were employed for the reference based mapping and alignment of the reads.

For the discovery and filtering of SNPs, Samtools 0.1.8^40^ along with in-house customized scripts were used with the following parameters seed length (-l) = 30, maximum differences in the seed (-k) = 1, number of threads (-t) = 16, maximum number of gap extensions (-e) = 50, mismatch penalty (-M) = 6, gap open penalty (-O) = 15, and gap extension penalty (-E) = 8. The merging and sorting procedures were performed using Picard 1.112 program and GATK3.1 was employed for genotyping and unifying the SNPs. The physical map of the selected SNPs was created using MapChart 2.3 ^41^.

### Computational annotations of filtered SNPs

The functional annotations of the SNPs have been carried out according to Lee et al.^27^. Briefly, the SNPs bounded sequences were fetched from the reference genome using BEDTOOL and queried against BLAST SNP-associated scaffold sequences were retrieved from the reference genome and used as BLASTn queries against the non-redundant nucleotide database at the National Center for Biotechnology Information (NCBI). The following BLAST parameters were employed for the search: E-value cut-off, 1.0E-5; word size, 3; number hits, 5; and organism, radish. The hits with highest similarity score and lowest E value for each SNP-bounded sequence were selected based on multiple hits, and utilized for the Gene Ontology (GO) annotations and pathway details using Blast2GO standalone suite (http://www.blast2go.com/b2ghome). The GO annotations were acquired by implementing the following criteria: annotation cut-offs of _ 55; weight, 5; and e-value hit filter, <1.0E-6. The GO enrichment analysis of flowering related SNPs was performed using PANTHER classification system. The SNP-associated enzymes and pathway information were retrieved from the Kyoto Encyclopedia of Genes and Genomes database using the Blast2GO tool. The impact annotations of SNPs have been performed using the SnpEff tool^42^ according to Bhardwaj et al.^28^. SNPs associated with flowering related transcription factors and genes have been screened manually and the interaction analysis of SNPs was carried out with STRING interaction database.

### SNP genotyping using KASP assay

For SNP genotyping using KASPar assays, 60 bp upstream and 60 bp downstream flanking sequences around the variant position (SNP) were extracted from the reference sequence (Supplementary Table 5). Subsequently, KASPar assays for the targeted SNPs were performed according to Cheon et al.^37^. Based on the fluorescence signal, the SNP allele call data are graphically illustrated for individual markers assayed using the SNPViewer. Further, SNP allele call data obtained have been utilized for calculating both pair-wise genetic distance and per cent dissimilarity matrix to construct a dendrogram using DARWIN V5.0.128 software (darwin.cirad.fr/darwin/ Home.php)^43^. Cluster analysis was carried out using the UPGMA method.

## Conclusions

The present study illustrated the transcriptome sequencing based SNP marker discovery in 33 inbred line of radish with diverse characteristics. The SNP markers identified were functionally annotated to gain knowledge on roles of SNPs and their effects in the functionality of genes. Moreover, SNPs identified were associated with flowering related genes and transcription factors which significantly varied among the early, middle, and late flowering accessions of radish inbred lines. In addition the KASP marker based genotyping of SNPs can be utilized for the genetic diversity analysis, germplasm discrimination, and seed purity test for quality control investigations which enhances the molecular breeding of radish. In future, SNP linked QTL analysis could be conducted for the identification of potential markers linked with flowering for the introduction of late bolting radish cultivars.

## Supplementary information

Supplementary table files

Supplementary data

Supplementary data 2

Supplementary data 3

Supplementary data 4

Supplementary Figure
