# Supplementary material for: Transcriptome sequencing assisted discovery and computational analysis of novel SNPs associated with flowering in Raphanus sativus in-bred lines for marker-assisted backcross breeding
Source: Hortic Res. 2019 Nov 1;6:120. doi: 10.1038/s41438-019-0200-0 (PMC6823433; doi:10.1038/s41438-019-0200-0)
Supplement: Supplementary file 1 — Supplementary table files [file 41438_2019_200_MOESM1_ESM.docx]

**Transcriptome sequencing assisted discovery and computational analysis of novel SNPs associated with flowering in *Raphanus sativus* in-bred lines for marker-assisted backcross breeding**

**Jinhee Kim^1^, Abinaya Manivannan^1￥^, Do-Sun Kim^1^, Eun-Su Lee^1^, and Hye-Eun Lee^1*^**

^1^Vegetable Research Division, National Institute of Horticultural and Herbal Science, Rural Development Administration, Jeonju-55365, Republic of Korea.

*****Correspondence: helee72@korea.kr; Tel.: +82-63-238-6674

**^￥^These authors equally contributed to this work**

**Running title:** Transcriptome sequencing and discovery of novel SNPs related to flowering in radish inbred lines.

**Supplementary table 1.** Phenotypic traits of 33 inbred *R. sativus* lines employed in this study.

| ID | Flowering | root length | root skin color | flesh color | leaf shape | Growth rate |
| --- | --- | --- | --- | --- | --- | --- |
| K01 | Early flowering | short | Green-white | white flesh, | lyrate shape leaf | None |
| K31 | Early flowering | short | purple skin, | white flesh, | lyrate shape leaf | None |
| K12 | Early flowering | very short | black skin, | white flesh | None | None |
| K15 | Very early flowering | short | white skin | white flesh | lyrate shape leaf | None |
| K19 | Early flowering | short | purple skin | purple flesh | lyrate shape leaf | None |
| K14 | Early flowering | very short | yellow skin | yellow flesh | lyrate shape leaf | None |
| K23 | Early flowering | very short | green skin, | green flesh | lyrate shape leaf | None |
| K20 | Early flowering | short | purple skin | purple flesh | lyrate shape leaf | None |
| K32 | Very early flowering | long | white skin | white flesh | undivided leaf | late growth rate |
| K33 | Very early flowering | very short | white skin | white flesh, | undivided lea | fast grown rate |
| K34 | Early flowering | short | red skin | white flesh | lyrate shape leaf | fast grown rate |
| K35 | Early flowering | short | red skin | white flesh | undivided leaf | fast growth rate |
| K36 | Early flowering | long | white skin | white flesh, | lyrate shape leaf | slow growth rate |
| K37 | Very early flowering | long | green-white | white flesh | lyrate shape leaf | late growth rate |
| K38 | Late ripening, | very short | white skin | white flesh | lyrate shape leaf | fast grown rate |
| K40 | Early flowering | middle | green-white | white flesh | lyrate shape leaf | fast grown rate |
| K41 | Early flowering | middle | white skin | white flesh | lyrate shape leaf | fast grown rate |
| K42 | Middle flowering | short | green-white | white flesh | lyrate shape leaf | middle growth rate |
| K43 | Early flowering | short | green-white | white flesh | lyrate shape leaf | fast grown rate |
| K44 | Early flowering | short | green-white | white flesh | lyrate shape leaf | fast grown rate |
| K45 | Early flowering | short | green-white | light green flesh | lyrate shape leaf | late growth rate |
| K46 | Early flowering | middle | green-white | green flesh | lyrate shape leaf | late growth rate |
| K47 | Early flowering | middle | green-white | green flesh | lyrate shape leaf | late growth rate |
| K48 | Late flowering | very short | light green-white | white flesh | lyrate shape leaf | middle growth rate |
| K49 | Middle flowering | middle | pure white skin | white flesh | lyrate shape leaf | fast grown rate |
| K50 | Middle flowering | middle | pure white skin | white flesh | lyrate shape leaf | middle growth rate |
| K51 | Very late flowering | long | pure white skin | white flesh | lyrate shape leaf | late growth rate |
| K52 | Late flowering | long | pure white skin | white flesh | lyrate shape leaf | late growth rate |
| K53 | Middle flowering | middle | pure white skin | white flesh | lyrate shape leaf | fast grown rate |
| K54 | Middle flowering | middle | green-white | white flesh | lyrate shape leaf | fast growth rate |
| K55 | Late flowering | middle | green-white | white flesh | lyrate shape leaf | middle growth rate |
| K56 | Late flowering | long | green-white | white flesh | lyrate shape leaf | fast grown rate |
| K57 | Very late flowering | middle | green-white | white flesh, | lyrate shape leaf | fast grown rate |

**Supplementary table 2.** Transcriptome sequencing statistics of 33 inbred *R. sativus* lines

| **Index** | **Sample** | **Total read bases** | **Total reads** | **GC (%)** | **Q20 (%)** | **Q30 (%)** |
| --- | --- | --- | --- | --- | --- | --- |
| 1 | K51 | 6,751,285,126 | 67,234,024 | 40.54 | 99.22 | 97.16 |
| 2 | K52 | 6,848,546,179 | 68,165,200 | 48.13 | 99.30 | 97.47 |
| 3 | K53 | 6,020,584,160 | 59,964,740 | 48.18 | 99.23 | 97.22 |
| 4 | K49 | 9,080,984,495 | 90,560,400 | 47.8 | 99.19 | 97.04 |
| 5 | K50 | 6,383,697,243 | 63,546,524 | 48.3 | 99.30 | 97.47 |
| 6 | K57 | 7,330,950,748 | 73,148,888 | 47.85 | 99.13 | 96.87 |
| 7 | K38 | 5,399,909,377 | 53,809,216 | 48.17 | 99.25 | 97.26 |
| 8 | K40 | 5,517,593,783 | 54,979,130 | 48.18 | 99.25 | 97.26 |
| 9 | K46 | 6,086,461,396 | 60,582,408 | 47.93 | 99.32 | 97.54 |
| 10 | K43 | 3,817,553,668 | 38,048,086 | 48.13 | 99.23 | 97.20 |
| 11 | K42 | 4,562,993,233 | 45,489,558, | 48.21 | 99.19 | 97.11 |
| 12 | K44 | 5,086,172,503 | 50,679,056 | 48.18 | 99.25 | 97.30 |
| 13 | K47 | 4,151,267,819 | 41,365,032 | 48.11 | 99.24 | 97.24 |
| 14 | K45 | 5,369,897,540 | 53,448,032 | 47.80 | 99.32 | 97.54 |
| 15 | K01 | 5,610,530,853 | 55,964,324 | 47.94 | 99.13 | 96.89 |
| 16 | K12 | 7,064,420,776 | 70,465,212 | 47.95 | 99.07 | 96.72 |
| 17 | K14 | 4,185,020,057 | 41,764,210 | 47.85 | 99.05 | 96.65 |
| 18 | K15 | 2,560,543,741 | 25,646,224 | 47.80 | 98.52 | 95.15 |
| 19 | K19 | 4,350,818,074 | 43,414,688 | 48.18 | 99.04 | 96.64 |
| 20 | K20 | 4,668,655,158 | 46,545,516 | 48.02 | 99.15 | 96.94 |
| 21 | K31 | 7,982,319,378 | 79,744,838 | 48.11 | 98.99 | 96.49 |
| 22 | K32 | 5,524,412,905 | 55,072,854 | 48.21 | 99.13 | 96.88 |
| 23 | K36 | 4,648,148,312 | 46,329,696 | 48.17 | 99.20 | 97.11 |
| 24 | K41 | 4,131,264,947 | 41,183,254 | 48.28 | 99.14 | 96.90 |
| 25 | K32 | 4,489,159,972 | 44,758,108 | 48.19 | 99.18 | 97.05 |
| 26 | K33 | 4,248,684,765 | 42,325,660 | 48.36 | 99.28 | 97.35 |
| 27 | K35 | 5,600,243,165 | 55,793,872 | 47.85 | 99.23 | 97.19 |
| 28 | K34 | 4,362,375,455 | 43,504,972 | 47.86 | 99.14 | 96.96 |
| 29 | K48 | 6,715,017,798 | 66,827,536 | 47.85 | 99.32 | 97.54 |
| 30 | K54 | 20,039,688,139 | 200,018,750 | 47.82 | 99.01 | 96.47 |
| 31 | K56 | 6,293,882,380 | 62,788,378 | 48.06 | 99.12 | 96.86 |
| 32 | K55 | 4,851,306,986 | 48,338,688 | 48.69 | 99.25 | 97.28 |
| 33 | K37 | 5,875,887,579 | 58,558,340 | 48.29 | 99.27 | 97.12 |

**Supplementary table 3.** Mapping results of 33 inbred *R. sativus* lines

| **Sample Id** | **Processed reads** | **Mapped reads** | **Multiple**  **mapped reads** | **Overall read**  **mapping ratio** |
| --- | --- | --- | --- | --- |
| K51 | 33,617,012 | 27,868,166 | 3,531,987 | 82.10% |
| K52 | 34,082,600 | 27,869,770 | 3,806,961 | 81.10% |
| K53 | 29,982,370 | 24,502,110 | 3,183,144 | 81.00% |
| K49 | 45,280,200 | 37,140,814 | 4,576,747 | 81.80% |
| K50 | 31,773,262 | 25,980,607 | 3,526,972 | 81.00% |
| K57 | 36,574,444 | 30,643,406 | 4,017,209 | 83.50% |
| K38 | 6,904,608 | 21,728,148 | 2,966,683 | 80.50% |
| K45 | 27,489,565 | 23,087,624 | 2,980,939 | 83.70% |
| K46 | 30,291,204 | 24,613,343 | 3,019,218 | 80.50% |
| K43 | 19,024,043 | 15,947,209 | 2,030,896 | 83.60% |
| K42 | 22,744,779 | 18,793,630 | 2,286,130 | 82.40% |
| K44 | 25,339,528 | 21,549,679 | 2,722,244 | 84.60% |
| K45 | 20,682,516 | 17,518,546 | 2,133,412 | 84.50% |
| K47 | 26,724,016 | 22,112,623 | 2,658,910 | 82.00% |
| K01 | 27,982,162 | 22,932,197 | 3,142,537 | 81.70% |
| K12 | 35,232,606 | 24,797,687 | 3,691,424 | 70.10% |
| K14 | 20,877,305 | 14,698,959 | 2,037,885 | 70.10% |
| K15 | 12,823,112 | 8,379,371 | 1,450,482 | 66.60% |
| K19 | 21,707,344 | 17,746,288 | 2,668,300 | 81.40% |
| K20 | 23,272,758 | 19,057,818 | 2,425,990 | 81.70% |
| K23 | 27,536,427 | 22,305,437 | 2,847,915 | 80.80% |
| K36 | 23,164,848 | 19,100,200 | 2,390,742 | 82.20% |
| K32 | 20,591,627 | 17,190,707 | 2,115,218 | 83.30% |
| K33 | 21,162,830 | 16,728,650 | 2,145,291 | 78.80% |
| K35 | 27,896,936 | 23,002,737 | 2,872,905 | 82.20% |
| K34 | 21,752,486 | 18,048,148 | 2,204,101 | 82.70% |
| K48 | 33,413,768 | 27,836,031 | 3,365,166 | 82.60% |
| K54 | 100,009,375 | 81,677,646 | 10,408,340 | 81.40% |
| K56 | 31,394,189 | 25,586,621 | 3,410,248 | 81.20% |
| K55 | 24,169,344 | 20,255,236 | 2,568,336 | 83.30% |
| K37 | 29,279,170 | 24,687,743 | 2,985,529 | 84.10% |
| K40 | 23,362,222 | 18,691,459 | 3,872,981 | 79.80% |
| K41 | 25,520,299 | 20,870,956 | 3,109,022 | 81.60% |
| **Average** | **28,683,155** | **23,825,281** | **3,135,957** | **80.89%** |

**Supplementary table 4**. MAB marker set details of the identified SNPs in Radish

| **ID** | **Chr** | **Genetic distance** | **Probe sequence** |
| --- | --- | --- | --- |
| NRS01 | R1 | 2713383 | AACAAAAAATGGTTTTTAACTGTGGTAATTTTATTATTTTACAGCGCAAGACAAGATTGC[A/G]GATTTTGAGATGAAGCTGATGGATATAGACAGTGAGCATTTGGGGATACCTGATGCTGAG |
| NRS02 | R1 | 2725564 | GACAGTTGAGGCACATTGTGGTTATCATTTCCCTTGGAGCCTCTCAAATTTTCTTCCTCT[A/G]TATGGAGGGTAAGATCACACACACACTCACACCTAGCAAAGCTCTTTTGAATCTAAAAGC |
| NRS03 | R1 | 2822960 | TTGGAACGTAATAAGAAGTTTAATTAATCCAAAAGTCCAGTCTTTTCCTCAGCATCAAGT[T/C]GTGACTTGACAGAGGCAGTATTAGGCCCGCTTGTAGTAGAGAAGCTAAGCGTTCTGCTAA |
| NRS04 | R1 | 3756966 | AGATGAGCTTACTAGCAGGTTTTGAGGCTTAATATCACGGTGACAAAGACCAAAGCTATT[A/G]TGGATGTAAGCAAGCGCCCTGCAAATCTGTAACCATTAACAGAATGAGACTACAAGAAAG |
| NRS05 | R1 | 4259232 | CATTGCACATTCAGAAGCAGCACCCGTCCGAGAAGCAGCAGGGTTTTGGGTTTATGTCTT[T/C]CAGGTCCAGTTCCAAGTAAGTCTCTTCTTTTTCTCTCCCTTAAGTCATCTTCTCGAGACT |
| NRS06 | R1 | 4283392 | CATTGCACATTCAGAAGCAGCACCCGTCCGAGAAGCAGCAGGGTTTTGGGTTTATGTCTT[T/C]CAGGTCCAGTTCCAAGTAAGTCTCTTCTTTTTCTCTCCCTTAAGTCATCTTCTCGAGACT |
| NRS07 | R1 | 4532795 | ACTCTGAAGGCTTTAGATGTTTCTTCAAATAATGATGTAGCTTCTTCGGACCTTGGAGAA[A/G]CACCCAATCCAGGAACTGCAAGAGAAGACACTGAGAAAAGAATACGTGCACTTAAGAAGA |
| NRS08 | R1 | 5337568 | TTAATAGTTCCCATGTTCTTGATCGTGATAGGTGGCCAAGATTGAGACTGAGAAGATGCT[T/C]ATTCAAATGGTTGAAACTGAATTGGAGAAAAGAAAGGAAGCTGGTTCATACAAGGGACAA |
| NRS09 | R1 | 5363263 | CATTGGATAGAAGAAGAGAGCACACATCTAAAATTAACATCCCTACCCTCCGTACAATGA[T/C]TGTGGTTTTTAGTCCCATCATCCTACTCTAAGATGGAAGATGGCCAGAGACGTGTCCACA |
| NRS10 | R1 | 6409284 | AAAAATCAAAAAGAAGTAGAACCTACCTTTATTCGCTGCATCACTGAATCTAGTTCTTGC[T/C]CACCAACAAGATCAGTCTGAAACAAAAAATACAGACATTTTTAGGTTGTACGATATGTCG |
| NRS11 | R1 | 6755104 | AACAGAGAACAGCGAAATAGAAACTTACAGCTCGATCCATGGCAAGTTCTACGTGTACAA[T/C]TTCACCAAAGTTACCTAAGAAATACGAAAAAAGAAAAGGCATTACTAACACACAGTGTGA |
| NRS12 | R1 | 6970201 | ACCGTAAGTTTGATGATCATGATAGTGGTTAGTCTCATAATCTACTACATACATACTCTG[T/C]TGCTGCTGGTAATAGCCGTGGGATGATGATGTGTCCTCCATCACCACCGTGTTGTTGTTG |
| NRS13 | R1 | 7351693 | GAAACATTCATCTGCAGCTTGTAAAGCAGCAACTGCCATGCCATGTGATTTCTCTGTGTT[T/C]CCTTCATCAAGAATCAAACCGTGATAGTAGTAAGCCGCAGCCTGATCCAAAATATTCCAG |
| NRS14 | R1 | 8215424 | TTTGCGGTTACTCTCAAAGGTAAGCGACCGAACGCATACATGTCCCCAGATGTCATAGCT[A/G]CCATAGGTTCCATGTACTGAGGTCAAATACTCATGCGGGTAATCAAGTATGACCTTAAAC |
| NRS15 | R1 | 8802756 | AGTATGAACCTTTCCGGAAGAACTTCTATATTGAAGTGAAGGAGATCTCAAGGATGACAC[A/C]AGAAGAAGTTAACGCTTACAGAAAGGAATTTGAGCTGAAAGTCCATGGAAAGGATGTACC |
| NRS16 | R1 | 8803219 | ATCAGGAGTTGCGCAGCAAATTAGTGAGCTGAAGCGAGGGACAGAGATTGTTGTGTGCAC[T/C]CCTGGGAGGATGATTGATATTCTTTGCACAAGCAGTGGGAAAATCACCAATCTGCGGAGA |
| NRS17 | R1 | 8835676 | ACAATCGAATTTCGATGGGAGAGATCCACGAGCTTGGTAACCAAAGAAGTGACATATAGC[A/G]TTGAACTTCTTCCCCGTGTATGTGCCTTCTTTCTGTCAAGTTTCAAAAAGCTTTATTAAA |
| NRS18 | R1 | 10418363 | AGATACGTTGGAGAGGTATTTGCCTAACTCGAGGGCCTTGGAGAGAGTGACGGAGATTGC[T/G]GCTCGAGCCAAGAGTGCTGGGTCGTTGTGGGCGTAGGTTGAGAAGTTTGAGAGAGAGGAG |
| NRS19 | R1 | 10650247 | AGCGCACGAGAAACTCTACATGAGGAACAGAGCGTTGAAGGAAGAGGTGAAACACAGATC[T/G]TATCTTCCGTACAAGCAAGTTGGGTTCTTCACTAATGTGAATGGCCTTAGCAGAAACATC |
| NRS20 | R1 | 11418985 | CAAATCAACAGGAACTCAGGGAAAGAGAGGTAAGTATCAGACAAACCTTCTGCCGAGATA[T/C]TCCATCAGACCATAAAGAACAAAGTGGCGATGTATGCCTGCACAGCAACATTAGAACATA |
| NRS21 | R1 | 14589102 | GGAGAGACCAAATGCAGCGAGTTTTGATAGTTATACCTGAGAAGCAAAGGTAATCTTAAA[A/G]CCAAACACTCGTAACTTTGCTTCTATACGTGGAACCTTCATTAGTTCCATGAAAAACTGA |
| NRS22 | R1 | 15846091 | GATTTACTGAACCATTGTCAAGAAAGGTGGCTCGGTATGGCCATTGCCTCCTCTGTTTGC[A/G]GGGCTCTGAAACGCAACCTCATAAGCCGCAGATTTCTCATCCTCGTCTAGTTCTTCATCC |
| NRS23 | R1 | 16350200 | TCTATGTGACTCGCATGTGGTTGTCACTCCTAGAGTACTAACTCTCGACCTAAAGGCTTT[T/C]GTCCCAAGGATGCTGTTCCCTGTTGCACTCTTTCCGTTTCCAGTACGGCCCACAAGAACT |
| NRS24 | R1 | 19736557 | AAGCGAGAGCTCCAAGCTCTTGAAGCGGCAAATGTACATGCAATTCTGGAAAGCGAACCA[T/C]TAGTTGATGAGGTTATTATTTTTTGTTAATTATTATTTTTAAGACTGTCTTTGCATTTGG |
| NRS25 | R1 | 21212976 | CACCAATTATTTATCGTAATCATCGATAGTGCTATTAAACATAATACACACAATAAAATT[A/C]ACAGGAATTGATATGTTAAATGCACAAATAATTGTGGACTCAACCTGTCTCATCTTCACA |
| NRS26 | R1 | 22297617 | GTGACAGACCTTGCATGATGTAATAGTTCCAAAGGGAGCAAAATGTTCTTTGAGCTTCTC[A/G]TCTGTGACAGACTCATCCAAGTTCTTAACATACAAGTTGGACCCCTGAGACTTGTCTGCA |
| NRS27 | R1 | 25169050 | CAACTCGGCAATGTTCTATGGTCTTCCTCAGTACCATCATCATCAGCCTGCTCCCACCAA[T/C]TACTATGCTCCTCCCTATGTGGCGTTTCAGCCTCCCCCAAGTCAGTTACCATCTTCTAGC |
| NRS28 | R1 | 25810447 | AATCCGTAGTGCTTGTGATGGATGGGCTCATCTTGATGTCAAAGTGGGTAACACATATTG[T/C]TGGCCTACGAATCTTGGATGGGTGATGGGACCAACTCTTATATTTTCTTGCTTTCTGACC |
| NRS29 | R1 | 25861336 | AACAAAAGGTTCCGATCAAAAAATATCAAGTACCTGAGTTAAAGTGATGAAAACATGAAT[A/G]GGCAAAGATGCATCAGAACCAGTAGCCCTCAGCCGGAATTGTGGATTTTGATGCCATGAG |
| NRS30 | R2 | 7645564 | AGCTTCCAGGCACCTGAAGAACAAAAAAAAAAGAAACAAAGGGAGACATACGCATCCTTC[A/C]AGACAGTATCATAAGACGTCTTCGTTCTGAAAGAGACAACAAACGGATCAAAATTGAGGT |
| NRS31 | R2 | 7798678 | TGTTTCCAGCTCATCAGAATTGGTACTTGAGAACCAGTAACATGTTGCAGGTTGTAAAGG[T/C]CATATCTCTTAGCTGGGTTGTTCAAGAGTGCAATTTGGTGGCGTGGGAGATTGCAGTAAG |
| NRS32 | R2 | 8475954 | CAACTCTTTCATAAATTGTTATTTTTGTTTTGGTTTTTTTTTTTACAGCTTGAACTGTTA[A/G]TAACTACTCTGATCGACCTCGATGCAATGGATGGTAAAAGTAGTGCATCACTATTAGCTG |
| NRS33 | R2 | 10898597 | ATGTAAACAATATTGAACAATACGATTCAACATACAATCAATCTCAGGCAATCCACTGAT[T/G]AGGAGCTCGAGCTCTTTATCATTAAAAATGGATACGAGCTCACGAGGTATCAACTCATTA |
| NRS34 | R2 | 18530239 | TATCTAGAAAACAGAGGGAAGTGTACTTAACTATTAACATACAAGCAAAAGATTTTACCA[T/C]GAGTAATGTTTTACACATCGGAGAAGTAGTTCGCGAACCGTTATCAAAGCATGAGTGACC |
| NRS35 | R2 | 18530733 | GACTACTTTGCGTATTAGTAGTACTGAGAGACGATCTCCAAAATGCCACAACTTTCATCA[A/G]AGTCTCTTCGTCTAGGTTCAAATTGACTGGCTGCACATTAAGAATATAAAATCCAAAGTC |
| NRS36 | R2 | 24558955 | GTGAGGGTTGTCCTCGGAGGGTTTGGGTTCGTCGAAAACGCTCGTCATTGTTTTTTTATC[T/C]TCAAGCTTCTTTTTCCCGTCCTTCACGATGGTTTTTTTCTTTCCTTGAAACCGAGGTGTC |
| NRS37 | R2 | 26179373 | AAGGCAGCTGTTGAAGGGGCTGAGAAGCTATCCGCTGAGCAGTTGGAATTCGCAAAGGAC[A/C]GAATAGTTATGGGCACAGAGAGGAAGACGATGTTCGTATCAGAGGACTCGAAAAAGGTAA |
| NRS38 | R2 | 26191046 | TATCAGATACCTGCCCACATGATAACACCGCCAGCGTTTTCAATTCGCTTTCTTTCATCG[T/C]TTCTATTAGGCTTATGGTCATCTGATAGCGCAATCGCTGCACAACATAAAAACACTAAGT |
| NRS39 | R2 | 28332784 | ACAGAACTGGACAAACCCACGCTTGCAGGTGGCAGAACCAGTGTTCTTGCCGCTCCATTT[T/C]CTGGTTGCAGGGTCAAAGAAATTCTCACCCCACAGCCTCTCCATCATCTTAGTCTCATCA |
| NRS40 | R2 | 28363633 | ACAGAACTGGACAAACCCACGCTTGCAGGTGGCAGAACCAGTGTTCTTGCCGCTCCATTT[T/C]CTGGTTGCAGGGTCAAAGAAATTCTCACCCCACAGCCTCTCCATCATCTTAGTCTCATCA |
| NRS41 | R2 | 31644052 | GCTCCTGACGGGGAAGTCTCCCGGAGAGCCGACGAACGGGATGGATCTGCCTCAGTGGGT[T/G]GCGTCGATCGTGAAGGAGGAGTGGACCAACGAGGTGTTCGATCTCGAGCTTATGAGGGAG |
| NRS42 | R2 | 31925760 | TGTTTGTTGTGAGAGATGTGAATTACTTACAAGAAGACCTTCTTTACGAGCAAGAAGCCT[T/C]GCCATATCGATGGATTCATCACTTGAAACCTGTAGAAAGCGTTACAGACAGAGATGGAGG |
| NRS43 | R2 | 32418161 | AGAGAAACCAAACGCCTCCCGCCGGAGACCGCAGAACCGGAGATTCCTCGGAGCTGCATT[T/C]GATGAACAGAGCGGACGGTTGGGTGGAGTAAGAGAGGACTGTGAGAAGGAGAGAGAGAAA |
| NRS44 | R2 | 32440659 | AGCCTTGGTGCGGGGAAGAGTGGGGTCAGAAGCGACGTCGGATAGGATCTGCGTCTGTTC[A/G]CTTACGGAATGATGAACCTCGTTTCTGTACACACAGTTGTTATCCGCCGCTTCCTGCAAA |
| NRS45 | R2 | 32447318 | CACTGAAGAATAAATAGGCTGCTGTAACTGTTGGCCCTGATATTGCTGGTAAACCTGTTG[T/C]GGTTGTTGCTGCATCGGCTGGGAATAAAGAGCCTGCTGTGATTGGGTCTGAGGCTGCTGT |
| NRS46 | R2 | 33107878 | GAGAGAGAGATGGAATGGACTTACAGCAAACCTAAGAAGCAAGCAGGACTTTCCAACACC[A/G]GAATCACCAATAAGCAAAAGCTTGAAGAGATAGTCACTGCAGAATCAAAAAGAGTTAAAT |
| NRS47 | R2 | 33427064 | GCTGTCTCCTTTGCCGAAGCCGCTCTTTTGCCATGCTCCGAGGAGGTAGAAGAAACAGCA[T/C]AGTCCGACCACGAGGATTAGAGATAGTGAGCTTCTCGATCTCGGTGGATTATTCTTAGAA |
| NRS48 | R2 | 33437619 | GCTTGATGAGATCTTGATTCCTACCCAACTATCTGGAAACGGGGCATGATTCACAATGTT[A/G]CGCAGAAGATCAGCAAGAATGAGCTGAAGCTTTACTCTGTCTCCATAGAGAGGCAGAGTT |
| NRS49 | R2 | 34465317 | TTGTTTTGGCTGTTCTTCAACAGGGAGAGACCGCACAAGGGCAGCCACCTCTTCAGCAGT[T/C]TTCCATTTCGCTAGCTGACCAAGACGCTTCTGACCAGCCCAAACGCTTGTATGGATAACC |
| NRS50 | R2 | 36800337 | ATACACAAGAAAATATTTCCACGTTTGCAAACCAAGGAACAGCTAAACTTACACTCTCCA[A/G]AACGAAGATATATTCCGGCCATCTAGAAATGATGACTTGATAGTCTTGAATGGTCTCGTT |
| NRS51 | R2 | 37300172 | AATCTTAAAGCAAATCAAGAAACTTGCCTTCGGTCCAAGAAACAGCCCATATGGCACACC[A/G]TCGAATTTGTCTGTGTGATGCAGCTGAATACACAATCAATTTTGTAATTAACAATGACAA |
| NRS52 | R2 | 37452812 | TTAACATTCTACATTTTTCCAGCTTGGTTACTTCTCATTTGGTGGAAGCACAGTTATATG[T/C]GTCTTTGAAAAGGTAAGCTTCTAACTTGCACCACTATGATAATTACTATATGCTTAAAAT |
| NRS53 | R2 | 39008624 | ACATATTCGTCTTTTCAAGTGCTTAAAAGTTAAAACAAAACACCTTATTAACTGTTGCTT[T/C]GAGAATTAACAATAGAGAGTGATTTATTCTCTCATTTAGTGACTTAACACTTTCAACCTC |
| NRS54 | R2 | 39012714 | AACAAAAAGAAACTCAATTTTGTGTGTAATTTACCGGTCAAGACGATGGCCTTGACATCA[T/C]TCCTCTGATCCGCGTCTTGAAACTTCTCCTTCAACCCAGAAATAACTGAATAATTCCCAA |
| NRS55 | R2 | 39016678 | CCCGGCCAAGAATGTCGGAGCTGGCCTTGACGACCAAAAGAACTTTGTTGCGTTTGCTGG[A/C]ATCGGTGGAGCTGCTGGAGTTGGTGGTGTTGGTGGTGTCGGAGCAGGTCTTGGTGGTGTA |
| NRS56 | R2 | 39027469 | TACCACTTGGGAAATTACTAGCCACGGACTCTAATGTTTTGATTGTGGTGTTCAGGTGAC[T/C]CATCCGGATTTCAAGAAAGCCAAAGAGAAGGTTATGTTCAAGAAGAAAGAAGGCGTCCCT |
| NRS57 | R2 | 39187252 | CAAAGAGAGAGTAAGGGAGAGACTGAGGAGAGCTCACCCATCAGTAGATGGAGCAATCAT[A/G]TACAGACAGTTACCATAGCCTGCAGTTTCATGAGAGTTTAAATGAGACTACAATGGCTTC |
| NRS58 | R2 | 39844114 | GTAAGACTGGCCACAACCCACGAACATAACCGGTGCTCCCGATATGTAAACCATCGATAA[T/C]GCTGCTCCAACCTACAAAACCCCGTCCCAAACACATAGTTTACAATTTTCATACTATAAC |
| NRS59 | R2 | 39915680 | TGTCATCCTTTGTTCCTTTTCTTAGGTGAATAGCCGTGTGAGTGAACAGTATGATCCTTG[T/C]ACGGAGAAACACTCCAAAGTGTATTTCAATCTCCCAGAGGTTCAAAAAGCCCTCCATGTC |
| NRS60 | R2 | 40448626 | TATAAGTATACTAAATGAAATTAAAATTCGAAGGAGCAGACTAGAATACAATGTAAAGTA[T/G]CCATCAGGCCTGAGGAAAGTAATATTCTGTCCACCATTGAGGACAACTTTGATATTTGAG |
| NRS61 | R2 | 40483948 | CTTCATGGACAAGAGAGACGGAGAGATCAAGGAGAAGCTGGCGAGCGTGAAGGACACATC[A/C]ACGGAGATCAAGGCCCTCGATGAACAAGCCGACTCTGTGATGAGGGCTGCTAGGGCTGAG |
| NRS62 | R2 | 40997850 | AATAAAAGAGAAGATTAGCAAGAGCAGAACCTGGAAAACTTCAGCACCAAGATAGCTTTG[T/G]AGCATGCGGATGACAGATGCACCCTTCCTGTAGCTTATTGCATCAAATATTTCATCAATT |
| NRS63 | R2 | 41149147 | GTAGGAGAGAAAGAAGCAAAACCTGATGGGTGTGAAAAGTGAAAAGGAACCGTTAACAGG[A/G]ATATCTCCAGTGAGACGAGTGTTTGAGAGATCCCTGGATTTTCCAAGATGATATCATTTT |
| NRS64 | R2 | 41499841 | CCTTGACTATGATAAAGAGGCTAATCATTGGATGCTTGGCAGCCCCGTATTACCATTCTG[T/C]TGAAGCGCTGCATCTATGACAGTGATGATGAGGTGCGTTCTTAGTCTTAGTCTAGAAAAG |
| NRS65 | R2 | 41500363 | TGATCTGCAGGATCCCTCTGAAGAAGCTTTTGACATCAACTCTGTGCCTAGGGAAGTAAA[A/G]TCCCAGCCCCTTGCAGAGAAGAAAGCCCAGGGTAAAAAGCCCACTGGTCTCGGTGCGCCA |
| NRS66 | R3 | 781900 | GTATGCATCGCGGCCACGAGCACGGAGATAATCTTGCAATCCCTCGTAAGTTGATCCTTG[A/G]CTTCTCAGCAAATCCGGAGTGAGATCATTCAACAGATAACAGTACCTGATAGAGGTTGGA |
| NRS67 | R3 | 1663104 | GCTTAACGTTTGTGTTTCTTTTTTCTACTTATAGATGCGTGGAGTCCTCGAAGTGAGACT[A/G]GCTGCAGCCCTGGAGATTAAAAATGCTGCTGAGAAAGAGATCCAAGAGAAAGAAGGTGCC |
| NRS68 | R3 | 2770726 | CCTTTAGACTCCAAACCAAAAAGAGTATATCACAAAAGAGGCTTGGGAAGTTCAATACCA[A/G]GAACCGGCAGGAATGGCAACAAACTCCTTGTTCTGCATCACTAAGAGATAGTAAGTTGCA |
| NRS69 | R3 | 5841257 | TTTTGACTCATTTCTATAGGAGATTGCATTACTTAGTCGGCTTCAGCATCAGAATATAGT[A/G]AGATATCGTGGCACAGCCAAGGTATGTTCACTTGCAGCTGCCTGAGAATTTTTCTTTGCT |
| NRS70 | R3 | 6861949 | TGAGGAAGGGCATAGATCAGAAGCTGAGATTGTGTCTTTGAGGGCAGATTTTGCTCAGGA[A/G]GTTGCTGTTTGGCATAAACTGGACCATCCAAATGTTACCAAGGTTCATATTTTACTTATG |
| NRS71 | R3 | 12187156 | GTTTTAACGCAATCTCGGTGGCCAACACTTCCTCTTGATTTTAAAAGGGATCAATACTAG[A/C]TTGTGGTTGGGGAGAATGAGAAGAGCTATGACAAAAGGTCAAATGTATGTAATTTTGGAA |
| NRS72 | R3 | 13258694 | TGCTGATTTCAACTGTTTGTTTTTGCATATATCTGCAGTAGATGATGCACATAAGACGAA[T/C]GGTTCTGCTAAACACGGTGCAGGTGAAGACGACGCAGAGGTAAACTAGAAGATATTTCCT |
| NRS73 | R3 | 13363778 | CCTGATGTTAGTATCTAAATATGGCTAAACTTTTTGTTTACAGTGGAACTTTCGTATTGG[A/G]TTCACTCCAAGGGAAGGTTTGGTCATACATTCGGTAGCATATGTTGATGGCAGTCGAGGT |
| NRS74 | R3 | 13567933 | TTTTGCAGCTAACTCATAACCAGTGAAACCATTATTGCGGCTTTTGTATAACCCAAAACC[A/C]TTCCATGAACACACTAAAGAGAGATCTGATCACTCACCAAAATAGTTGTATATATGGCAC |
| NRS75 | R3 | 13577222 | AGAGACCAAAAAAGAGTTCACACAGCGGGATGTCCAAGAAAAGTTCGACGTCACTCTTAC[A/G]AAGCATAGGGGACAGGTGATCTATATGAACCTTGTAACTCTTTGTGTGCAAGCATTTTCT |
| NRS76 | R3 | 15585278 | ATGGCCACAGCTTGACTTCAAAGAACAGAAGGTGGAGACTATCCCCAATGTGGAAAGCAA[T/C]GAGTTAGAAGAAAAAGAAGAAGAAGAAGTCTTAAGCAAAGGTGAACAACATATGGTGGAG |
| NRS77 | R3 | 16026573 | ATAAGTGACATCAAAGGGCTGCTTGATTCAGCTAGAGTTGATCCAAATGTGAAAGGTGGT[T/C]TAAGGTGGCCGTTTGGTAAATCTTCATCTGGGAATGGATACAGTGTCTTTGAATCTTGTC |
| NRS78 | R3 | 16074996 | TTTCATACCTCTAATCTTTAAAACTCTCTTTCTTCTTCTCTCAGATCTGCCAAACTCTGC[A/G]CCAACAACAACAATGGCTTCCGCATCTCCTCCCACCCTCCCAATCTCCAACCCCCAAACC |
| NRS79 | R3 | 20832921 | TGGCTACCTGTAACGTGTGAGGATTTTAATTATTTTTGGTTTTGTGTTGCATATGGCACA[T/C]GCCTGCCTGTCTGCCATAAGGACGGCGCCGCTTTGAACCTGTCCTCGATTTTTGTCTATT |
| NRS80 | R3 | 21711190 | GATAAAAAAGGAATGCTAGTTCCTCTAATTGCTACTTGGAAAGCCAAATACCTTCAGATC[A/C]GAAGAAAAGTTTCCAACGGTTTTCTTGTAGCCACCTTTCTTGAGATGGAAGTTCAACCAT |
| NRS81 | R3 | 21891360 | GCCTCCTCCTCCTACTCCTCCTCTCTTAGCTAATAGCGCCTGTTGGTTCCTATTAACCTG[A/C]GCCTCCCCCATTCTCTTAATGGAATGAGGATTGCCAAAGGCAACAACACAAGGCCTGCCG |
| NRS82 | R3 | 23881075 | GTATTGTATAGATTTATGTTTGCTGAACCTTGAATTCTTGTGCATGTAGACAACTCTCTC[T/C]GAAGCTGGACGGAATTTCAAACTCACTCTTATTGCTAGGCGTTCCCGCCATAATGCTGGA |
| NRS83 | R3 | 23882590 | AGATTGTGATATCAAGCCTGGCAGGCTCCAGAGGGGCGTGCTGAGGACCAATTGCATAGA[T/C]TGCCTTGATCGTACAAATGTTGCTCAATATGCATATGGTTGGGCTGCTCTAGGACAGCAG |
| NRS84 | R3 | 24345136 | TGCTCGTCCCTCTTTTATCATATTTGCAGCCAAGTCTATCATAGCTGGTGTTTCAGGAAG[T/C]GTTGCTTTGTACTCTCTATCGCTGACCGGTGCCTGAGTAACCACAATGCAATTATGCAAT |
| NRS85 | R3 | 24500729 | AAGCAACAAGTTTTATTCATTTGTTGGCCAGGACATCACAACGGCATTGGCAAATGACCC[A/C]AAGCTTGTCTATCACTGTGGAATAACTCCACGGAAGCTGCCGGTATGTGATTCCATTATT |
| NRS86 | R3 | 25044585 | TTGTTAGTTTTCGTAGAAAGTGTGTATATGAACATTGTAGTTATTTACGTCAAGATGTTT[A/C]AACGCAAACCCGAAAAGATCTACAAATGGGAGGCAATGCAGGAAGACATAGAGGTCGGAG |
| NRS87 | R3 | 25340919 | TCAACAGAGAGGTAGACAAAACATACCAACTTGAGGTTTCACAGAGAAAATCACAACATC[A/G]CTTTCTTCAACCACCTGTCAACATAGTTCATTCATGGATAGTTTCTATCTTAAAGACTAA |
| NRS88 | R3 | 25874183 | CTATTTGTGTCTGATTTTCGTGTTTTTCTTTTGCAGCTTGTATAAGGGTCTTGGTCCACT[T/C]TGGGGACGTCAGATTCCTTGTAAGTTCTCGCTTATATTCTGAAACCTGTCTGTTGCACAA |
| NRS89 | R3 | 26159368 | GGACCGGTTCCCATTTGGGTACTTGGCTTCCCTTGTGCTGAAGAAGTACCTCTCCTTTTC[T/G]AAGTTGCCTGCATACAGAAAAAGAGGGCTTCATAAGTCTTAAGCTTGAATTTGAGATTTG |
| NRS90 | R3 | 27020189 | TGTGTTGAGCAGCAGCAGCAACAGCGATGATGCTGAGATTGGTAGGAAGGAGTTGGGTAG[A/G]TGTCAGAATGTGAGATCGTTTGAGATGAGTAGATGCTCCAATGTTGTTAACAAGGAAGAA |
| NRS91 | R3 | 27282763 | AGATCAGTCCAAAGATCCCGTGACAGTGAAGAATCTGAGATCGTACGAAGACACTTGCTC[A/G]CTAATGGCCTAGGCGTCACTCCTCCAATGGGGTCCGTACAACTCTTATTTATCTCTCTCT |
| NRS92 | R3 | 27287490 | ACTTCTTTTTGTTTGGTTATTAGCATGACAATGATAGCAGATCAGAATGATAGATGGGCG[T/C]CTTACGCAAGACCAGGATCATGGAACGGTAAATAACTTTGTTTAACTAACAGTTGTGAGT |
| NRS93 | R3 | 27287927 | GTAGTAAGTAAGAGCTTCAGCTGTTTATGTTCAGACAAACTTGGGATCCAAGGAAAGAAA[A/G]TCAAGAAAGAAGGTGATCTTGAGGTATATATACTTCTTTTTAATATATGCATGCACATAC |
| NRS94 | R3 | 27640730 | TTTGTCAAGCATCCAATGCTTGGGAGCATTGAGCCTCTTCAGATGCTTCTTCAATCCTCT[T/C]GCCTGCAAAATATTACACAATGATCAAAAACAGACACAGGCCAAACAGACAGAACATCAA |
| NRS95 | R3 | 28259354 | AAGACGACATTATGAAAGAATAAATGATCAAGTGTGGGTTTGGGGTTTTTACCCGATAGA[A/G]ATGAAAAAGCCGAATATGAACATTGAGATGCTTCCCGCCAAGCTCTTTCTTGGGTTGTAA |
| NRS96 | R3 | 28952187 | ACCAGCGGTTCCAGCTAAGAAACCTGAGATCATGGATTGCCAAGGGTGCAAGGATTTACC[A/G]TCGCCTTCGTGTTTGTTCCACAAGAGGACGTCAAAGGAGTTTTTGGCAGTGAACATGACA |
| NRS97 | R4 | 20594 | AAAAGGAGTGAAGAACACACTTACCTTCACATCATTTAGCTTTATAGGTTCCAGGTACTG[A/G]CTGAAAAACTGTCCCAAACTAGACCCCTGCAAAATGAAAATGACAAGGTTATATGAATAA |
| NRS98 | R4 | 40088 | AAAAGGAGTGAAGAACACACTTACCTTCACATCATTTAGCTTTATAGGTTCCAGGTACTG[A/G]CTGAAAAACTGTCCCAAACTAGACCCCTGCAAAATGAAAATGACAAGGTTATATGAATAA |
| NRS99 | R4 | 87867 | ACCCATGGATCGTTAGAAGGAGGCATTCCATGATCAACAGAAAGCGACTGGAGAGCTTTC[A/G]CGATGCAATCACTGAGTGATGCTTCCTCGCAGCCGGAGACCGCTGAGAAGTAGACGGAGA |
| NRS100 | R4 | 146571 | AGAGAGAGGCCACTCTATAAATCTGTATCGCAACCGAGTCTGTGAAGTGTGACTGAGGAA[A/C]AGATCCAGGTTGCTCTGTCGTTTGGCCCCTGACAACTAGAGCAGTTGAGATTGATACAGC |
| NRS101 | R4 | 156827 | ATCCTGGATCTGGCTACTCTTGGAACAACTCTCTGGGTTATATTTATGATCCGTTTTAAG[T/C]TAAGGCCTAGTTACATGCAGGACAAAGACAACTTTGCTCTCTATTATGTGGTAATCAGTT |
| NRS102 | R4 | 1718426 | ACGCTTGAGAGCCTGCGTGCGAGGTATCACCGACTCGCCTACAACGTCCTGCATCTCCGG[T/G]GTCACCGGACGAGGCTTCATTATTCCACGTGGCTCCCTCGTCTTGGTGGTTGACGACGGC |
| NRS103 | R4 | 3016596 | TCTTCCACCTACTTTAGGTGGCTCCATCTCTGGAGAAACACCGCCGCCACAGTACTGCAA[A/C]ACCGGAGAGTTAAACGAGTTGATTCTCTCCGCCTGCTTCACAGCTCTATGGTCCATCCAA |
| NRS104 | R4 | 3020654 | CCTGTAGGAAAGCCAATCACTCAAGTCCATCCACTTATACACCATTGACCAAGACTCCTG[A/G]AGATTCTCTTTCACCCATAGTAACTGCAAAAAGCAGAGAGATATAATCGTCATCATACTC |
| NRS105 | R4 | 4538199 | TTACATAAATGAATGTCTTGGAATGCTACTAACCTTGATTGAGATAACAAAGTGACCTCC[T/C]GTTTTGAGGAAGAAGCTTGAGTTCAAAGCCACGATCCTAGCCTATTTAAGAGGTTTATTT |
| NRS106 | R4 | 5426582 | ACAAAGCAGAGATTCGACGTGGTTACGCACGATGGAGTCACCAATGAACGCCCATGATTT[A/G]TTCCTCATAAGTTGAAGAAACCTTCGAGGATCAAAACGTGGTAACAGACAGTCATGAGGC |
| NRS107 | R4 | 7152682 | CTTTCAAGTCTTCCAAGGCCTTTCTCATATGCATCAGCGTGGTTACTTCCACCGCGATCT[T/C]AAGCCAGGTAATTGATAGCCTTGAGGTGGCAATTTATATAAGCTATGAACAGTTGCTGAG |
| NRS108 | R4 | 12854516 | CACTATAAAGTTAATATCTATGCATGAGACAGGCTGTACCGTCAGTGAGTGCTCCACTTT[T/C]GCGGCTGCATCCAGAAGACGCTGTTCTTCTATAAAAGGCAGTTTTGCTATACCCTTTACA |
| NRS109 | R4 | 12998076 | ACTTATGTTTGGCTCGTTTCCAGACTGTTTTTTTCATACCTGGCAACTTTGGAGCATTAG[A/C]GCTGGTTGCTGCTTTGGAAATTGAGTTGGAAAGAAGAAGTGAACCTACATCAAACGCCAA |
| NRS110 | R4 | 15163882 | AACCTTAGTGATAACGCAGATGTCCACGTTGCTACCACTACCCAGGTCGTTGAATATACC[A/C]GAGCAAATGGCCTCAGCGACCAGCTTAATTCCTTCATCCCTCTATAGAGGATATTGTAGC |
| NRS111 | R4 | 15862901 | AAAAAAAAAAGCATAAAATGAAAATAAAATTGCATTCACTACTGACCGTAGCTCCTCGTC[A/C]ACATAGGTAATCTCTAGTTCACCACGTGCGGTTTCAGGTGCATTTACAGGGATCTGCACA |
| NRS112 | R4 | 17860904 | CGATCCTGTGGCGCTGAAGTTGCTTCAGCGTTTAGTTGCTTTTGACCCAAAGGACCGTCC[A/C]TCTGCTGAAGAGGTAAGTAGAGCAAAAGCTGAAGCTCAGTTTTTCATTCATTTTCATGAT |
| NRS113 | R4 | 18391527 | CAATCAGGAAGAATAGTTCGTTATTCAAAGAGAAGTGGAGAATATGTAACCTGACGACCA[A/G]CGGGTCGGGTATTGTTTAATAAAAGTGAAAACGGAAAAAGCAAGTCCCTCCTGTACTCCA |
| NRS114 | R4 | 18829502 | TAAGATGATTAGCTTCTGCAACCTCTATAACTGCAATGTCGACATTCTCCTTTGCAAACA[A/G]AGAAAAGGCTATCCCAGTGAGAATCTGACACAAAGGAATATATCTAATATAAGAAAACCA |
| NRS115 | R4 | 19121843 | ACATCAGAGGCCACCGTTGAAACCAGTCTATGAACCTGGCATGGGTGCTCAGCGTCTTAC[A/C]CAGTATATGTACCGACAACAACATAGGCCTGAAGTAAAGCTTCTTCTTACCTTAAAATGA |
| NRS116 | R4 | 19124498 | AAAGATTAGGCCATAAGCCGTTACGCTATGCTGTGCTTAAGAAAAGGAAGAGTTAAAAGT[T/C]TTCCTACAACAACTGGTTTCTAGGCCACCCCTCGTCCACTACCACAAATACCATCTCGAG |
| NRS117 | R4 | 19287553 | ATTCCAACCCAGAAGAGATCAGAGATGAACTTAGTCATTCCCACTTTAGCAATCGCGTCG[T/C]TGAAACCATGTTTCAATAGCTGAGGCCCTTCGACCTGACCATAAGATGAAACCAATAACC |
| NRS118 | R4 | 25434861 | ACTCTCGTGGATCCAGTGCCATCGCAAGAAGAAGAAACCACCAACGACGTCGTTTGATCC[T/G]TCGCTGTCTTCATCCTTCTCCAACTTGCCTTGCTCTCATCCTTTCTGTAAACCAAGAATT |
| NRS119 | R4 | 26567553 | GAAACAAAGGATTGCGATCGCGAGGGCGCTTCTTACAAACCCGAGAGTCTTGCTCCTTGA[T/C]GAAGCAACAAGTGCACTCGATGCTGAAAGCGAATATCTTGTTCAGGTAAACAAAAACATA |
| NRS120 | R4 | 32241045 | CCATCTTGAGCACAGGACTTTGCAGAAGATTCTTCTGGTTGGAAACAGCGGTTCTGGAAC[T/C]AGCACAATATTCAAACAGGTAAATGAGCTTCTTCTTCTTCTGATTGATTAAGCCTAACCT |
| NRS121 | R4 | 32652494 | TTCTGATTTGATTTTCAAACAGCCAAGGCGTGGTTGCTTTGAGATTCGTGAGGAAGGTGG[T/C]GAAACGTTTGTCAGTCTCTTGGTAAGTCTTCCATTTGAGCAACTTTGTTCCCTTTTCAAC |
| NRS122 | R4 | 32674003 | CACAATTCATTTCTTTGTGGTTTCTCAGTTGGGACAGCAAGTTGGTGATGCCACTCAATC[A/C]CATACGCTCAACGCAAACCAGGCAACCACATTGAACCCTGTAAGATGACATATACCAAGT |
| NRS123 | R4 | 35113315 | AACGACAGACCCCCGTACACCCTAGTTAAGCTGTACAACACTTGCAGCATCCCCTTACAC[A/C]ATCTGAGGCTGGTTTACCGACTGCAAACCCATTCTCCTCTCTGCACAACAACATAACACC |
| NRS124 | R4 | 39217350 | GATACTTTTGGTCTCTTTGCTAAGTGTTACCAATGTAAATCACCGGTTCCAGCTGCCGCC[A/G]CTCATGCTCCGGTCTCTGCTTTACACCACCGCCTTTGAGAGATCTAGCCAGCATATATAA |
| NRS125 | R4 | 43597012 | CACCTAAACCGAACAACACACTCAGAATGTCGCAGGAACCCATAGACCAGAAGCAATCAA[T/C]AGACACATCTCAGCACCTGATACAATGGAAAATATCAGAATCGAGTTATATAAGCTAGGG |
| NRS126 | R4 | 43609092 | CACCTAAACCGAACAACACACTCAGAATGTCGCAGGAACCCATAGACCAGAAGCAATCAA[T/C]AGACACATCTCAGCACCTGATACAATGGAAAATATCAGAATCGAGTTATATAAGCTAGGG |
| NRS127 | R4 | 45045013 | AACTGCGAATTTCTCCTCCTCTCTTCTTCGTGTCTCAACCGCATCTTCTTCGAGTTCAGG[T/C]GTTGTGGCGTCGTCTCTTGAAACGGCTGAGAAGCGGGAAGGCGTAGATTGGTAACTCGGG |
| NRS128 | R4 | 45220680 | TGAGAAGCAGCTTCAGAAGATCACCGATGATAATCGGAATCGAGACCAAACTTGACCAAC[T/C]ATTTTCTTGACTTTTGTTGGAACATGAAAGTTTTGGAGCCGCTAAAATATTTTTCGCTGA |
| NRS129 | R4 | 45312715 | ACCAAACTTACCTCTCTGGTAGATTCATTGTGTCATCATCAGATCCTTCTTTGGAACTTC[T/C]TTTCCCTATATCTCTTCCTACATATTCTCCTGCTTTAACCGGCAATGGGCGTGGAGACAC |
| NRS130 | R4 | 46084665 | AACTCCCAGACTCCACTCTCTCCTACCTCGACCCCGCCACCAACGACGTCAAAACAGTCA[T/C]CGTCTCCTCCCTAACCGCCGGGAAGAAAACCATCATCTTCGCCGTCCCGGGAGCCTTCAC |
| NRS131 | R5 | 272206 | TCGTCTTTTTTGTACGGATCAGGTGGCTCGTGCTTCCGCTTTCGGGCTGGGTCTCGTCTA[T/C]GGCAACATCAAGCTCAAGGCCTTGAAGGTACTTCTGAGAGTTTTAATATTTGAGATTAGA |
| NRS132 | R5 | 277562 | TTTCTCGACATCCCCTAAATAAAATGCGTTTTGATTTGTGCAGGTGAGTCATGTGTACTA[T/C]CCAGGCCTTGCAAGTCATCCCGAGCATCACATTGCCAAGCGACAAATGACTGGTTTTGGA |
| NRS133 | R5 | 506306 | TGAGGGGCTTGTTCTGGATCATGGTTCAAGACACCCTGACATGAAGCGACGTGCAGAGAA[T/C]TGTCACATCCTTACTTGCAATGTGTCTCTGGAGTATGAGAAGAGGTGTGTAACAGAAACC |
| NRS134 | R5 | 3148228 | GTGAGATACCTTTTTGGCATCTTTTTCATAAACAGAATAGAAGAGATCAAGCAATCTCGT[T/C]CGAGTAAATGTTTTGATCTCTCCCATCATGCCAAAGTCATAGTAGATGATTGATTCGTCT |
| NRS135 | R5 | 3409056 | TGAGTTTTAGCTTTCAACCTTTGAGGGATCAAGTCCAGGCTGAAAACTGAGTCCAACCAC[T/C]CTTTCAACTTTCTGATGAGGCCGAGTAGCAAGGACAAGCCTTCAGATATGAAAGAAAAAT |
| NRS136 | R5 | 3964216 | GAAGAACTCCCTCTGTCTCCTCCTCTCTATAGGCCACAAGTCTCTCCACACCTCAGCCGA[T/G]AGCTGATCTCCTTTAGTAGAACCAAGGTACCTCACTATAGCGTTTGCAACTATCGGCGCA |
| NRS137 | R5 | 4036547 | GCTACAGATTCTAGGCTCTTATGGATGGGAACCATCTTCTTTCACAGCTCTTGCGCAGCA[A/G]TACGTGCGCATGAAGAAAACATATGGTGGTAAAGTGGTTCCTGGAGCAATGGACACAGCT |
| NRS138 | R5 | 4042820 | AATATCAAGACTAGTTTAACAGTTAAATAGCTCACCAAATATCTTGACAATAGTATCTAA[A/G]ATTGAGTCAAGAAGATCTTTTGCTGCAGCCTGCGCTTTGCCTCCAGGAGCCATAAGAGGC |
| NRS139 | R5 | 4043679 | GATATGTTAAGTGTAACAAGCACGTACGATAAACTCGTCAGGAGAGGAATCAGAAAGCCA[A/G]GGTGGAAGTTGGTAAGAAAGCAATTTACTATCAGTGCCATTCCTGATAGTCTGTCCATCT |
| NRS140 | R5 | 5955320 | ATCCGCTGGTCATATTTTGACATGTCTTTGGACTATCTCTTATCCAGGATCTGCTTATGG[T/C]GGCTTAGACTCTCCTAAGATAACATTCCGACAAGGTGTGAATCTAAGAGCTGGTTTCAAC |
| NRS141 | R5 | 6530552 | AGAGGGCGCACATAAAGCTTTAAGCCTTGGTGGAACGATGCTCGGGTACTATCCCGTGAG[A/G]GTCTTACCCTCCAAAACTGCTATTCTTCCAGTGAATCCCACATTTCTTCCTAGGGTAACC |
| NRS142 | R5 | 7993789 | GGAGAGGCTCGAGAGATCTGTAGTGCGGGTCCAAGCCATGTTCAGGTCTAAGAAAGCTCA[A/G]GAGGATTACAGGAGGATGAAACTCACTCATGAAGAAACTCAGGTACAATTATGTTTTGCT |
| NRS143 | R5 | 8734018 | AGATGGGGAATGCGAGAGCGAAGCAACTAGTGCTGCTAAAGAGAGGCGGCCAAAGAGGAG[A/G]AAAGGATTAGAGAGATGCTCAGGAGATGCATGTAGACAAGAGGATGAGACTTTCTGTGAA |
| NRS144 | R5 | 12682616 | GTTTAATAAACGCTTATTCTTTCTTTCTTTGTAGGATCATCCTTGGTTCGTAGGTGTGGA[A/G]TGGGGAAAGTTATATCAAATGAAGGCTGCTTTTATTCCCCAAGTCAATGACGAGTTGGAC |
| NRS145 | R5 | 16873288 | AGTCACAATACAGTGATTTGGTTAGGAACAAATTACTTACATACTCAAGTCCACCCAAAG[A/C]AGGATACTTCCAGAAGATCATCCCAAGATCAGCAATGAAGTAACCAATGGATAACTGCAA |
| NRS146 | R5 | 19361198 | TTTCCAGTGTCGGCAATGGAATCACCGAAGCTGATGATGGATTTGAAATTCCGGCATTTG[T/G]TTTCCGAGTTGACGATAGTGAGAAAAAGAGTAGATAAAAAGAAACTAAGGAGCTTCTTCA |
| NRS147 | R5 | 24237825 | TGGTTTTGCTAAAAGCCTTGCCCTTTTTGTTGTGATGGATGCTTCAGGAAAAAGCCATGT[T/C]TGTTGCCTCTATCCCAACCAGAACTCGGAACTCACGTTGACGGTTGAAAACGCTTTTCGT |
| NRS148 | R5 | 24456572 | GCAGTCTTTTCAAATAACTGACTAAGAAACCTGCTTCAAACTTAGGTAGCAAACTGAGAA[A/G]GCCATCCAACGTTGCGACCTTCTACGGGGCTTTACTACGAAGTGAGATAACACCCACCTT |
| NRS149 | R5 | 25338280 | GCATTATAATAGCTACCAAGTCTAAAATGGATGCTGATATAATAGGAAAATGGTGGTGAT[T/G]GGGCTGAGCTTGAGGTTCCACTGCCACAATGTTTCATTGATACCATAGGAGAGACAAAGA |
| NRS150 | R5 | 25763676 | GTGAGATGGTTTGATTTCGTTTTGCAGAGCGATCATTTCCCGTTTATCCTCTGCGTTGAG[A/G]GTTTGGTGACTGAGCACAAGTACGACAAGTGGGAGACTTGTTATGAGAAGCTCAATCTTA |
| NRS151 | R5 | 27053178 | GAATTCAAAACTAACCTCCATTAGCTTGGTGAGCAACTGAACTTTCTCACGATTCTTCTC[A/G]TTGAATGTATCCAATGCTTCTTTGTACTCCCTTTCCTGTTTCATGTCATCCACAAACATC |
| NRS152 | R5 | 27571696 | CTCTTCAGGAGTCTTGTTAGCTTTCTTAATCTCGTTCGGCCTCCTGGTCTGAACCAAGCT[A/G]CTCTCAATAGGGTCCTCAACCATCCTCCTAACGGGGAAAAACCGAAAGTTATCTTCCCCA |
| NRS153 | R5 | 27628953 | GAAAGATCTCTTGCTTATCTTGGTATAAACCCAATTATTATATCTTTCAAGGAAACATTA[A/G]GAAGTATAGTGTTTTTGACTCAAAGCTTGGAAGGAGGTTTGTACCATCCCCATGGTTCAT |
| NRS154 | R5 | 27953537 | TCACAGAGACCAACCTCAGACATCATACAGGGACCGTGCTGCAGAAAGAAGAAACTTATA[T/C]GGTTCATCAGCTCCAAGTGAAAATGATGTTATGGGTTCAAGTAAGCGTCCTTGGGTCTTC |
| NRS155 | R5 | 28429365 | CACAGGGATGCCAGAGACACTATTACCATCAGAAGACGAACCATCCTGAACATCCGTCCT[T/C]TCAACATCATAAAACTCGTCTTCAGAATCATTCTCAGAAGCTCCCTTGGTAGATTTGGCA |
| NRS156 | R5 | 30074145 | TGACTTCTCTCTGAAACTTCCTTTTTTTCTTTTGCACTAGATCCAAGTGTTTGCTGATAA[T/G]GAGACCTTCAAGCGAATGGGTAAGTTGCTGCTTATTGATATACAAGTCATATATATAAGA |
| NRS157 | R5 | 30416909 | AATCTCAGGAGTTATGCTAATGCTTCTCCTCCTTATTCTCTTTGTTTCCAATTTCGCTCC[T/C]CCAACAGATTTAGAGCTGCATATGAATACATAAGACTATTTAACAAACTTAAGGCACACG |
| NRS158 | R5 | 31641574 | GAAGACTCTGCTACTGGTTGGCTTCCTGCTTTCTTCTAAAGCAGCTTCTAGCTCACTTCG[T/C]TTCAACTACGTAGTGAGGAAAAAGCAGGTCAGATTAGATCATACATTGACAAGGAGGTAG |
| NRS159 | R5 | 34352551 | TATACATCTCAGGAAATGATCCGAAGGATTCCGCTTCTTCGGGCAAGGAAACCCTGCTTC[A/G]GCAAAGAACTGGCAGAGACAATAAAACGTCAGCGTTTAGCGATCAGACAACCGCAATTCT |
| NRS160 | R5 | 35680762 | AATAAGCTGAGGGAAGCTGTGTTTGAGTCCCCAATCTTGGCTGTAAACTTGTGAATCTCC[A/C]AGTTCTTGACAGCATAAGCCATCATCATCAACACAGATTCCTTTCCTATTCCTTTACCAC |
| NRS161 | R5 | 36305250 | AGGGTTAGAGGTATCACTGAATCTGCCAGAGAGGATGATGGAAATGGTGGTAGGCACAAA[A/G]GTGTAAGAAGCTCCGATAACAGCAGGGAGCCTGGTGCCGAAAAGAGATTGGAGCAAGGTG |
| NRS162 | R5 | 36832603 | CATGTCATTCCATATGTACAAGGACGGAGTTGAACCAACATAGTTCTTATACGAGAACCT[T/C]CCACCCCACCATTCTCTAATCTCTGGACTCAACATATCAATGTAAGATGATGAACCAGGC |
| NRS163 | R5 | 37085711 | ACCACTTGGACCAACAATAGCGAGAATCTCCCATGGTTTGGCTCTGCAGGTCACTCCTTT[T/C]AGTACATGCTTGACTTTCTTGCAGGTTCTTCCATCCTCCAGCTTCCATGTCTCTTCCTCT |
| NRS164 | R5 | 37737746 | GAGCAGGAGGAGGTGATGAAGAAGGCGCAGAGCCTCGAACCATATGAACGGTGTGATCAG[T/C]CTGCAAACCTAAGGCACAGTTCACAAGAGATTAGAAACAGTAAACGCTCATGAGACAAAT |
| NRS165 | R5 | 37895188 | ATTTTAAGGGAATCTTCTCTTTTTTTTTCTTTTTTTTTTTCAGTGGATCCCAGAGCTGAC[T/C]CACTATGCCCCTGGTGTCCCTATTGTTCTTGTTGGAACCAAACTAGGTTTGTTCCTTTGC |
| NRS166 | R5 | 37895911 | CTTTTACTCCCTTACTTAAACCTAACTCGCTTAACCATTGGCAATCAAAAGGGAGAGGAA[T/C]TAATGAAGCTAATTGGAGCTCCTTCCTACATTGAGTGCAGTTCAAAGTCACAGCAGGCAA |
| NRS167 | R5 | 38319929 | CATTTAAGGGAGGATAGGGGTGATGGGGTCAGGTTGAAGAGCTTTGTGAACAAGATCGGT[T/C]TGCTCCTTAACATGAAGCTGGTAAAAACCAGTGGCTGTAGCAGCTGAAGGAAGACGCCCA |
| NRS168 | R5 | 38388718 | GTGGTATTGTCTACTCAAACAAAGTTGTAATAATGCCATCTTCACCTTCTGAGGGAAGCA[T/C]ACTCCATCGTAAATCAATCCCTGGTCTCGAACCAACTTTAGCTGTTCACAAGTAAGCTCC |
| NRS169 | R5 | 38895737 | AAGGTTGAGACTTCGAATCTAAATAATATAAAACCTAAAAGAGCTGAGGAAGCATACCAA[A/G]CAAGGTTTCTTTAAGCTGTTCCAGTGATCAGTTTTTCACGTTGGTCGAACTTCTTGACTT |
| NRS170 | R5 | 40048146 | GATGGAGGTGCAAAACCCAACAAGGCAGATACTGATGCAGATACCTCAGAGAGCGACATG[T/G]GAAGCGCCTGTTGTGGATTTCATTTCAAAGTAAATTTAATCAACAACTAATTTGATTCTA |
| NRS171 | R5 | 40291346 | GTTCAAGTTGCTCTCCATGTCCTCTGAGAAGCTTAGACGGAATGGGATCAGTCCCCATTG[T/C]AGGTTCAACCGTCTCCTCACCGAGGTTGTGGTCGTGAACGCGAAGATCGGGCAGTCAGGG |
| NRS172 | R5 | 43041462 | ATCGGCACGGAACTCCTCATCCATATCATCTCCCACATCTTCCATCTCATCATCTACGTC[T/G]TCCATGTACTCAGCCGGAGCATCCCCTTCGTAATTGGACATTTTCCTTTTGTCAAAGTAG |
| NRS173 | R5 | 43863891 | AATGTTCTGATTAGGTGTGATACGAACATCGAGTTTGTACTTCTCAATGACTTCTCTCAG[T/C]GTCTTCTTCATGTTACCACCAACACGTCCACTGTCTACATGAAGCCCACAAAACCAAGCA |
| NRS174 | R5 | 44014753 | GTATACCAGATCCACGGCCCATGAACCCATATCAAGAAATCTCCCAAGAAGCACAAGAGC[A/C]CGAAACCGATGGCATTGGCTAAGTAACACCTGAAGCAAATCAAAGTCTATAAAATACGCT |
| NRS175 | R5 | 45344258 | AAGCCTAACGCTCGTGTCATAGACATTTTGCATTAGGGTTTTCTCTCTTGGCTGCTGCAG[T/G]TAAAGCACCGAATCTTTCAACCCCAACCATGGCGACTCAACAAGCTAAAGCATCCGTGCA |
| NRS176 | R6 | 49476 | TCTGTAAGATTCTTTTGGATTGGTTAATGGAATCTCTCTTTTTACTTTACAGAGATTTGG[T/G]GTCAAGCAGAAAGAAAAAATTGCATCTTTCAAAACGTCAGTGGATGTGCTTACCCTCTCC |
| NRS177 | R6 | 3780188 | TTGTGTTTTGTCTTGATGTGCAATTTTTCAGGCTCGTATTAAGAAGATTATGCAAGCTGA[T/C]GAGGATGTTGGCAAGATCGCTTTGGCAGTTCCTGTCTTAGTCTGTGAGTCTTTTTTTTTT |
| NRS178 | R6 | 4089152 | CACATGAGGATCTGTGCAGTTTGGGTTTTCAAACGAGAACCTCTGGTGATTGATGAGCCT[T/C]CTAACTCTTTCCTCTTCGCCAGGCCTTATTGGGATCATGTCATACTCTGAAGCTGATGTT |
| NRS179 | R6 | 7730212 | TCTCAGCTTTTAGACAAGCAGTTGTAGAAATTGAGGATTATCGTCTATTCTGCAGATTTG[T/C]CCTGTATGCAGCGCAGAAGTTTCAACTTCCTCAGCTGGACAAAGCTGAATAGCAAAGGAA |
| NRS180 | R6 | 10972812 | TAGGGTGAGACAAACCTCTGCAGGCCAGGCATCTGACTCCTTGAGGCTCACATTAGTAGT[T/C]TCCAGCTGCTTGAACTCCGGATAAATACATGCACTAGAAGCATAGAAGAACCTGAAAACC |
| NRS181 | R6 | 13196709 | TCAGGCGAGCACTTTTGATATGGTTCTCGATGGTGCTGATCTTGTCGGAAGGGCTCGTAC[T/C]GGTCAGGTATTGCTTTTATAACGCATTGGTCTCTGTAAAGGTCGAGGCTTTATAGTGCTT |
| NRS182 | R6 | 13555321 | AGACTGTAACCCCATTCACCCAAAAGCACCTTGCTTTAACCCTGACAATGTTAGGTCTCA[T/C]TGCAACTATGCAGTCAACAGCTTCTTCCAAAAGAAGCGTCAAGCTTCAGGCACTTGTGAT |
| NRS183 | R6 | 14736894 | TCTCCATGACCAATCTGCCAAGTTTGTATGCTCGTCTCCGTTGTTTCCTTCTCTTCCAGT[A/C]ACCAGCTCTAGCAAAACTACCCCGAAGCTGTACACATCGATCTTCTCATCCACCTTTGAT |
| NRS184 | R6 | 15210346 | GATTCGTCAACTCTGCTCCAACTCCCGTCAAATCTTCCTCTCTCAACCCAATCTCCTCGA[A/G]CTCCACGCCCCAATCCGCATCTGCGGTAATCTCTCTCTCTTTCTCCCACGATGAAATTGC |
| NRS185 | R6 | 16810880 | TTTATGTAAATACTTGATTTGGTACTAGGAATGGAAGAGGCTTCACATGGAGAAATTCAT[A/G]GAAGCGGCCAAGAAATTTACGGAATGGAAGAAGAATCCGGATGGGCAATCAAGGGAGGAG |
| NRS186 | R6 | 17817973 | AAGTCTCGAGATCATGAGTCTTGAGAACTTCTTCAGCTGCTTCTCTTGTGGAAAAGACAA[T/C]CACGGGGACCACGCCGAATCGAAGAAACATCACGGGTCCATAATCTTGAGAGAGTTTATG |
| NRS187 | R6 | 18794695 | TGATGAGCTGAAGGTTAAAGTGAATAAACTAACCTCCATCAAGACCCAGCTTCCCTACTC[T/C]TATTACTCCCTTCCTTTTTGTCAACCTAACAAGATTGTTGATAGTACTGAGAATCTTGGT |
| NRS188 | R6 | 19173408 | TGTATTACATCATCGATCGCAGGGATTTTGAACTATTTTAATGTTTATCTGCAGATCATT[T/C]CTATGCGGTGGGTCGACTGGTTTGTTTATCTACGCGTACTGCTTATACTATTACTACGCG |
| NRS189 | R6 | 23655454 | ATATACCTGTTCACCAGGATTCAGACTGTCGAAATCAAGCAAGTGGTCGTACTCTGTCAA[T/C]GGAAGATGTGATGACCTGAAGTTTCCCCACTCCATGTTGATCACCTACATTGCAGTACAT |
| NRS190 | R6 | 24604213 | AGGAGCGCTTGGTTTTACAGCCAACGTCAAGCCAAGAAAACATTCATCTTGTTTTGTGTT[A/G]AAGTCCCAGAGGTTTAGCTCAAGATTAGGCATCTCCGCTGCTGAATAAAACAAAAAAAAG |
| NRS191 | R6 | 26982437 | CTTTATACTGTAGTCTCTGAATGCACAATCGCGGGCTGCACTCATGATGAAGCTCGATCG[A/C]AGTGGAACTGCATCAAGGTTTCCCTGGCTCCTGTTACTTATTTCGGTTAATTTTAGTTCG |
| NRS192 | R6 | 27612070 | ATCTTCAAGCTCGTTCTCTGGGTCCATGTTCTCCCATGCATTCCTGAAATTTGAAACACC[A/G]ACGTTCACCATGTAGTCAGCAGGGACAATCTCAAGGTCCTCGAGCTGGTACTCATCTTCC |
| NRS193 | R6 | 32560826 | TTTTTTTTTAATTTCCTTTTAATTTGTTGTTAACAGAGGGATGACATATCTTCACAATGA[A/G]CCTAATGTTATCATTCACCGAGATCTTAAACCAAGGTAATCACTATTGCATAGCTCTTCG |
| NRS194 | R6 | 33352153 | AAAAAGAATGTGATATACGAATTGTAAGAGTCATCACCTCATCGTATTCCTTCTTATCAG[T/C]TTTTGGCTGCTTCAAAGGTTTCGCCTTTCCTCCTGTTTATTACAACGATAACGATTAGCA |
| NRS195 | R6 | 33432703 | AGAAATCGGGTCGGAGCGTATTCAGAAGATAATTGATGATATGGTTAAAGTAATGAGATT[A/G]GCTCCTGGAGTTGGCCTTGCTGCTCCTCAGATTGGTGTCCCCTTAAGAGTAAGTAAGCAA |
| NRS196 | R6 | 34953931 | GTTAAGCGTCGTCGCTCCAGCTTTTATCACTTCTCCAAGAATCTCATACAGAAACTCTCT[T/C]TCCGATCTAGTCAACAATTTAAAAAAAGAAAAAAGTTAATCAAGATTACAACTAGTTAAA |
| NRS197 | R6 | 36007915 | TGTTCTATGTGTCAAGAAAGGGCTGAACATTGAGTAGATGACACTGAAGTACCAAGGCAC[A/G]TTGATGAATATCTACAGAAATTAAAATAAAAAGGATTCATCAAAACTTCACGAGTTGAAA |
| NRS198 | R6 | 37428825 | AAAAATATTAGCAAGGCAATAGAAAGATGGAAAATAATATCTTACATTGACAAAGCAATC[A/G]TGGAAGTGAAGACGAAGAATGCTCGCGGCGATACGAGGGTCCGATCTTAGCTCGTTGATA |
| NRS199 | R6 | 38452960 | TTTGAACTTCCCACGCCAAGAACCATCAGGTTTCATTTCAATCTCAGTCAACTCTTCATC[A/G]AGATGTCTCATCTGCCAAGAAGGAAGACGTCTTTATAAATTTGCTAGATTCATTTACTGA |
| NRS200 | R6 | 38604252 | CCGTCCTAAATCTAAGAGAAACCAGAAGAAAGGCAGGGATTCATCCTCGTCACAGAAGTT[A/G]GATTCGAAAGCAGGAGGCAAGAAAGAGTCCGTTAAGGCGCAAGAAGGTAACAACTTTATC |
| NRS201 | R6 | 39168702 | TCCATTCTCAAACTGTTGCAGGAGATTCGAGTGTTCTTGATGGGGAGGCGAGGGTTGTTG[T/G]TGGTGAAGGATGATAATCTTTTGGGAAAAGGGGAGATTTTGTAATTTAGAGAATCGTGAG |
| NRS202 | R6 | 40173148 | CTACGAAGCCGAGTCCACAGATAATGAAGCACCGGGACAAGGAAATCAGACGGGAAGAAG[A/G]CCATACCGAAGAAGGGGCAGAGGTATTACCAGTTTCTTAAAATTTTAACGAAGTAAAAAC |
| NRS203 | R6 | 42313725 | GCAAGTGCTGAAACAACAGCGAAACCTGAGGCTGCCACTGCTGCTGCTGCTGCTGCTGCT[T/G]CTGGCTCGGGAGAGGGGGAAAAGCCTGTGGCTGAGGCTGAAGGAGCCAAGGCAGAGTGAA |
| NRS204 | R6 | 42377967 | GATAGATGGGCAAAAGCTAATAGTTGCTAACGTTGGAGACTCACGGGCAGTCATGTCAAA[T/G]AATGGTGTTGCATCTCAGCTCTCTGTTGATCATGAACCAAGCAAGGAGCAGAAGGAAATA |
| NRS205 | R6 | 42630052 | GATAGGTAGGAAAAAAAAAGAAGTCACTAATTATACCTGAGAAATGTGCTCAGCTGCAGC[A/G]ACCCTTGGCTTAACAACTTCGCAATTCGCTATGACTAGAGTGTTGGGCACACTGCCATCA |
| NRS206 | R6 | 44616358 | GAGAAGACGGTGGAGCTGATAGAGACGCACAAGGAGAAAGTGGCTGAGATCGCCGAGCTT[T/C]TGCTGGAGAAGGAAGTGCTGCATCAGGATGATCTTTTGAAAGTTTTGGGTGAGCGTCCGT |
| NRS207 | R6 | 44861564 | CTGTTTGATCCGGTTTCCTTGTGTATACAGCTGCAGGATAACATCAGTCTCTTGTATCAG[A/G]CAAGAAATAACATCAGCGCCATTCTGACCGAGTATGTGTCATTGGGTCTTCTTTTCCTTT |
| NRS208 | R6 | 45346613 | AAAACGGCTCAAGCCTTACCTCAGTGAGTACCACAAGTAGTCGCTTTTATCAGCTGTTGT[A/G]TTTATCTGCTCTAACAATCCAGGTTTCACGAATGCATCAGCCTTGGAGATTCCAACAGGT |
| NRS209 | R6 | 46522277 | ATCTTTGCTTGTAAACGATGTTCCAGGAGTCCTTAATCTTGTAACTGGTGTTTTCGCTCG[A/G]AGGGGATACAATATTCAGGCATAGTCCTAATCTCTCTCCCACAACCATACAAAGTGCGCT |
| NRS210 | R6 | 48373198 | AGAGACAACAATGGCGGTGAAAAGCAAGAAGAAGAAGATGGTCCTTAAGGCAGTCTCTGT[T/C]GTAGACATCGGTTGCAGCAACTGTAAGTTCCCAACCTTGTCTTCTTTTTTCAACCGTTCC |
| NRS211 | R6 | 48800918 | GTCAAGATCCAGCAATTACACTACTTTTACCTTGAACAAAGTTGGTAAGACTGTTTGAAT[T/C]GTCAAGAGAGCAAGACCTATGAATCCTGTAACAGCGTGCGGACTGCATTGCACAACCATA |
| NRS212 | R6 | 48859791 | ATCACTGCTCGCCTTCACACCGGCTCTTGCCGTCTCCGACAACTCCACCTTCACACCATT[T/C]CCAACGGTGGAGATTGCTGCCACTTGTCCGATCGTCAGAGTCTCTCCTCCTAGATTCACC |
| NRS213 | R6 | 50064392 | ATGTGTCACGCAGAGATGTACGCCAACCCATCGCCTTTGTGATCTCTTGAATTTCATCCA[T/C]AACATCAATTAAATCCCTTATGTATGCTCGCCCTCCCGGTCTCAATATCCGGTCCATCTC |
| NRS214 | R6 | 52266567 | TGACAAACAAACATGTGTATTCATTGGGTTTCAGGTTGTGCAAGAGGCACTTGATAGAGC[T/C]AATTTGACTGAGAAGGATCTCTCTGCTGTTGCTGTTACCATTGGACCTGGTTTGGGCCTC |
| NRS215 | R6 | 52753864 | AGAGAAAGAGAACCGTGCCTCATTGACGCTCCAAAGCTTTACCGAACAGTCATCACTTCC[A/G]CTGACAAATTTTGTCGGGTCAGATGGAGAAAAATCAACTGACCAGGCTCTCTTCTGGTGT |
| NRS216 | R6 | 52798338 | GGGACTTCGGTTGGGTTACAGCTTGGACCAGACGGAGGAGCTGGAAACTCCAGAGACAAT[A/C]TGAGGCCATTTGATCAGATTCCTTGGAACTTTAGCCTCTCTGATCTCACTGCTGATTTGT |
| NRS217 | R6 | 53290592 | CCTCTGGCTCATATTCTTGACCGTATGAATGAGGAATACTTCTTTCGCAAAGGGGCTTCC[A/G]TTGGCTATTACCATGGGGTATGTTAATCTCTTAAGACACTAACATTCATCAAATAATGGA |
| NRS218 | R7 | 225671 | TTATTTCTTGTAATACTTGCCTCTTTCTGGTTTAACACAGGGTGCAGTGGACACAACTAC[T/G]CTAGAAACTCGTATCAAAGTGGTGAATCCTGAAGATCCAGAACCATTGATTAATCTCTAT |
| NRS219 | R7 | 226586 | CGCATGGGTATCAGAAGGAAAACAAGTGGGAGATGAAGTCGAAGTTAGAGTAGAAGAAGC[T/C]CATCCCCGCGACGATCTTATTCTTCTCAAAGAAGTTCTCTAGAGAGTTAACAACACCTTA |
| NRS220 | R7 | 506517 | TCCTACTTATTCTTTCATTCAAACAAATGGGAATTAAAAAAAAAACAAGTTACCTTGCCA[A/G]TGTTAAGCTTCCCAGGTAATTCCGAAGATGAATCAGAAGCAGGTTTTGAAAACCTAAAGA |
| NRS221 | R7 | 514867 | TCCTACTTATTCTTTCATTCAAACAAATGGGAATTAAAAAAAAAACAAGTTACCTTGCCA[A/G]TGTTAAGCTTCCCAGGTAATTCCGAAGATGAATCAGAAGCAGGTTTTGAAAACCTAAAGA |
| NRS222 | R7 | 542986 | CATTAGCAGTTAATCGGATGCAAATAAATTCAAGATAGGCTTCAAAACTTTACCTTCATA[A/G]CTACAATTACGAAGAAGCGCAGTAACGATGTAACGAGTACAGTCATCATTCTCCACCAGC |
| NRS223 | R7 | 1323542 | ATACGTATCTTGTGGTGTAAGTAAGTAACCATCCAATGGGTTGTTGTTGTTACAGGATGA[T/G]GATGATCAACTGCGTCGATCTCCTGGGACGCCAGTCTTCACTGCTCCAGAATGTTGTTTA |
| NRS224 | R7 | 2672570 | CCTTAAAAGTTTTTTTGCTTCACCATGGTTGGTCATGTCATTTAACTGTTTGCAGTGCCC[T/G]GTTCTGATTGCGTGGGGAGAAAAAGATCCATGGGAGCCAATCGAGCTTGGACGAGCTTAC |
| NRS225 | R7 | 5410047 | AAGTTTTTATTTATTTATTTTGATAGGTAAACATGCTGGAACTGATAAAAGATTGTCTCT[A/C]AGTTTGGAGAATGAGGTAATTTTATTGTATAATTACTTGCATCTAATTATTATTACAATC |
| NRS226 | R7 | 5552215 | GTACTTGATGAAGTCGATGCAGTTCCCAGCTACAAGCATTGCTGGAAGGCCCCAGTCTGG[T/C]GCTTTAGCACATGAACCAGGTGAAACAGGATCACTTGACCCGTTGATTGGAAAGAAGGTA |
| NRS227 | R7 | 5560027 | GGTGGATTCGAGTCAGGGTGGTGCTTGGCATGGGGGAATGGTCCCTCTCGAACAACAATA[T/C]CAGCTGTTTGCATCTTCAGGTGCCATTAGATTTCCAATTGAACCAGTGACAGAAGCTTGG |
| NRS228 | R7 | 5563323 | CATTTTGTAAAAGGGCTTGAGATGATGCTGCTGCTCGTTGTGTACCAAATCTTCGGAAGT[T/G]CTTACAGAGGCGTGCTTGCGTATCTTCTGATCACCATATCTATGTGGTTCATGGTCGGGA |
| NRS229 | R7 | 5563590 | GAAGAGCAAGAGCATCTCAGACATTCGGGAAAGCGTGGCATAGTTGTTGAGATTTTGTTG[T/G]CGCTGCGGTTCTTTATCTATCAATACGGTCTTGTTTATCATCTCACCATTACTGAGAGGA |
| NRS230 | R7 | 6666036 | CATGTTATATACAGTCTACCCACATATGAGGCATCGCATGAGTTCCAGACAAAGAAAAAG[A/C]GGCAAGAGCAGCGTCAAAGAGAGGAAGCAGCAAAAAGACAGAAAGTGCATCATCCACAGC |
| NRS231 | R7 | 7741733 | TTTCCAATGCTTGCTGCTTTATATTCCTGTGTTTTTGGGTTTCCGTCAGGTAAAAGCTGT[T/G]GAGTTAGCAGGTTGTGATTGGATTCATGTTGATGTGATGGATGGTCGTTTTGTTCCCAAC |
| NRS232 | R7 | 7767467 | TTTCCAATGCTTGCTGCTTTATATTCCTGTGTTTTTGGGTTTCCGTCAGGTAAAAGCTGT[T/G]GAGTTAGCAGGTTGTGATTGGATTCATGTTGATGTGATGGATGGTCGTTTTGTTCCCAAC |
| NRS233 | R7 | 7817468 | CAGTGACTCAGTATCAGGGGCAATGGAGTACTACTCTGCCTATGTTAAAGATGTTCATAC[T/C]GAGAAAGATGTAAGACCTCTCTCTCTCTCTCTCTCTAAGCTCTTTTGTCTTTTTGAGATT |
| NRS234 | R7 | 8508169 | TGTGATGAAAGGTGTGTAAAGAAGCTTTACATTGTCTTGATCAACATCACGCATCATTTC[T/C]TCTATGCGGAAATCCTCAACACCAAACTCCTCACAAGCTTGTTGAAGCTCGTCCGGTGTT |
| NRS235 | R7 | 9468533 | AGACGGCGTTGGTGAGGTAGAGAGGGAGCGGGAGCGCGTCGGAGGCCTTTGGAGGAGAGG[T/C]GAGAGTCTTGCGACGATCGTCGTCATCGGAACGGTTATCGAGGAACCGGTTTGAGTTAAC |
| NRS236 | R7 | 10312888 | CTTTGTACTTACCTTTTTTTTGGTGGTCATGTATTAGGGATGGATGGGGCCAAACTATTC[T/C]ATCTCAACTGCTTGTGCAACAAGCAACTTTTGCATTCTGAATTCAGCAAACCACATTATT |
| NRS237 | R7 | 10350778 | CTTTGTACTTACCTTTTATTTTCTGGTCATGTCTTAGGGATGGATGGGGCCAAACTATTC[T/C]ATCTCAACTGCTTGTGCAACAAGCAACTTTTGCATTCTGAATTCAGCAAACCACATTATT |
| NRS238 | R7 | 12125285 | GAAGAAAGCTCAAAGGGAAAGGGAAGGTGGCCAGGGGGGATCATCTGATGATGATTTCGA[T/C]AGGAGTGGTGGAGCTAGAAGAAGCGCCGAGGACAAGATCAAAGACAATCTGTTTGATGAT |
| NRS239 | R7 | 17935701 | AATTTCACTCTTCAGGGTGGACAACGCTGATCTCGCCTCTCTCGAGCAAGTCGTAGAAAG[T/C]TCTGGTTTTCCTCTGCTCGTTCCAGTGGTGCATTTTCCAGAGACCACCAGCAGCTAAACC |
| NRS240 | R7 | 17943376 | TCCGTGCCACGGTTTGCTGTCCATTGGGTACCAGAATGCAACCGACATTTTGGCTGTTGA[A/G]CCAAGCGTGAACTGAACATCCTGTAGATAAGAATATGTTTTATCTTTAAACACATGTTAT |
| NRS241 | R7 | 26548778 | CTTAATCCCTGCTTCCTCTCCAGCCGACATTTCAACAATTTTAGTCTTGTACCGCTGCTT[T/C]TCCCCCCATCTCATATACATCCTAAGCAGCATGTCGGCCCAATCCTGCACTGGAAGTTAT |
| NRS242 | R7 | 26677088 | AAGCCCGAACCAAACCACTGGAGTTACATCGACTCCTGCAAGTGGCGGAATCACTTTCCT[A/C]GTCTGAACAAGGATTGGTTCGGTAGGAGCGTACGCCAAAACGTAAGGGAACTTGTCAACG |
| NRS243 | R7 | 26677340 | CAAGGCCTCCGAGATCGGTTTTGACGAGATAATACTTGAATAGAGGCTTGTTGTTGCTGT[A/G]TCTACTTCGACTTGGGTTAACGATGAGGAAACTACAGGAAGACATGCAGACCGAGTTCGA |
| NRS244 | R8 | 302278 | GTTAAGTTGGGTTAATGTAAAAAAGATAATCTCTGGTAGGTGAACTTCTTTCTTCTACGG[A/C]GACTGTTGGGAGTGAGAATAAAGGGATCGGAGAAACAAGGGAAAGTGACAAAGCTAACAA |
| NRS245 | R8 | 344815 | GTTAAGTTGGGTTAATGTAAAAAAGATAATCTCTGGTAGGTGAACTTCTTTCTTCTACGG[A/C]GACTGTTGGGAGTGAGAATAAAGGGATCGGAGAAACAAGGGAAAGTGACAAAGCTAACAA |
| NRS246 | R8 | 3969979 | AGTTGTAAAGCCCCCCAAAACAGTGAGAAAGCTTGCAAGAGTACAGCATTTATATTCTCA[A/G]TTGGCAGGGCAGCTAAGAAGAGAAGATGACGAGGTTAGATTTACGGTTCTTCAGTGGTTT |
| NRS247 | R8 | 4010251 | CTATGCCGAGACGGCAAATGGTACCGAGAATAATCAACTTAGCAAATCAAACGGCACAGA[T/C]CAAGAAGAAGTTGAGGGTGTAGTAGGTAAAAGGCGTTTCCTCGACCTAAACGAGCTTGCT |
| NRS248 | R8 | 4448138 | GTCACCTATAAACGGTCGATAATGAGCTTCCTCGTTCAAACACATTGCGATTATCGCAAT[T/C]GCATAGTTTAAACATCGTCTGGGGTATTTTCCTCGTAGAGAAGGATCCACTAAGTGTCCA |
| NRS249 | R8 | 6077701 | CGCCTCGCTATGACGCAATTTGCTTCTATCATCTTTGTTTCTTTCATCCTTGTTTCTATC[A/G]TAACTCCTAGTTGTTCCTTTATCAAGCCCCCTCTCAACTGAGCGCTCTCTACCATAGCGT |
| NRS250 | R8 | 6185623 | TAACTGCGCGTCGTTCTTTACTTTAGGTCTTGTTGGGTCAGTGCTTGCTAAAGAAGGGAG[T/C]TTACCTGATGTATCCCATTTTGTCTCTGGTGCTTTTAAGGTATCTAAACTTTTATATTAC |
| NRS251 | R8 | 7764468 | CTTCCCCGCCTGTTGATATGGAAACAGAGGAGGATTCTACATCAAAGAATCCTTCTGAAA[A/C]CCCGAGTGAACCAGAAGCCGAAAAATCACCAGCTTGGGTCGAATGGAGAGAGACATCCGA |
| NRS252 | R8 | 7791593 | GACCAAGAAGATTGTGCTGAGGCTCCAATGCCAAACCTGCAAGCATTTCTCTCAACACTC[A/G]ATTAAGGTAAAGAGGGCATTTTTTTACATTTTGCTGCCCAGTTATAATAAGTTTTGAGTT |
| NRS253 | R8 | 7993625 | CCAAACAGATGATACAGAAGCCGCCATGTTCTCTCTTCTTCTTTGCTTTTGCTTTGTGTG[T/C]GAATGAATGAAAGGTTGTGTCTTGTTTTCGTGAGAAACCCTTGGAAGCACGGATCCTTTT |
| NRS254 | R8 | 11371303 | ACTCCAAACAATTGATCTCTTTCACCCAGGCGTCATCAATCTCGCCTTCCCAGTCGATCA[A/C]AGCCCCGTCTTCTCCCACCACGTTCTTGATGGGATGCGTATCGAAGTACTCCGAAGCTCT |
| NRS255 | R8 | 11397853 | ACTCCAAACAATTGATCTCTTTCACCCAGGCGTCATCAATCTCGCCTTCCCAGTCGATCA[A/C]AGCCCCGTCTTCTCCCACCACGTTCTTGATGGGATGCGTATCGAAGTACTCCGAAGCTCT |
| NRS256 | R8 | 12423385 | TATATTATTTCAAAAGAGTAAAGAAGAAGAAGTGCTACCGATCTTTTCTTGCGGGGTAGC[A/G]ACGCATTTGCACGTATTGACCCGTTTTGCCATTGGTAAGCAAGCATTTCGAGTTCTGTAT |
| NRS257 | R8 | 14366315 | CTGGATGTTTGATACATAGGCGTTGTATGCAAGCACCTCAATCTCATCAGGAATGAAAGG[T/C]GCAACCACAGCTATGGTTGCGATGAACAAGGTACTGATCCCCACTTTGCTTCATCATTTG |
| NRS258 | R8 | 15608591 | TCAGGCGATGCACCACCGCTTACCTTTCGGCGCTCTCTCACCGCATCAACCCCAACCCCA[A/G]CATCACCAGCAGCAGCATCCTCACCATCATCAACCTCAGCCGCAGCATCAGATTGATCAG |
| NRS259 | R8 | 16763721 | GAACGACGAAAACATCCTCGTACCTCCTCCCACTGAATCAACCATCGACTACATCGCTTC[T/C]GAAAATCTCAAACCGTTTCCAGATCCAGAATCCTCTGTTCAGGTGTCAGATCCTCACCTA |
| NRS260 | R8 | 18438177 | CGTCGAGGTCATAGCACCAGTGATTCCAAAATCGAAATGAACCATTAGGAGCCATGACAA[A/G]AACACGTGGACGGCTAAGGAGACTGCAGCCACGTAGGCAATGATCTTGTTCTTGCTCTGA |
| NRS261 | R8 | 19009784 | GTATGCAACAAACTCGCTCTATAGTGCATGGGATCGTCAGTTTTACCCGGAACTCATGGA[T/G]AAAGGATCACACATAATACAGCTTGATGTTGATACAGAGAAAGGTGGTCTCTCCATAAAC |
| NRS262 | R8 | 20065885 | AATATCATCTCATCTTCCTCCGTGATTTCTGGTTTAGCTTTTTGCAGAGAAAACCCTTGC[T/C]TCTCAGCGATCGATCTGAAGAATTTCATCAGGTTAAATTTCGTTATTCAATAACCTGTTT |
| NRS263 | R8 | 21342116 | TCAAGTCGTTGGTTACCCAGCAATGATCAAAGCATCATGGGGTGGTGGCGGCAAAGGCAT[T/C]AGGAAGGTTAGCTTGTGTTCATTATTAGCCTTTTGACTCTCACTCTCTTCTTTTCATTGT |
| NRS264 | R8 | 21345046 | AACAGATGGAGATATTGATCCAATATTTTATTTACAGGCTGGTGAACTTATAGCCAAGCT[T/C]GATCTTGATGATCCTTCTGCTGTAAGAAAGGCCGAACCCTTCCATGGAGGTTTCCCAAGA |
| NRS265 | R8 | 21346976 | AGCTCCTTTATCTGGCAATATGATGCACATTGCTATTGTGGGCATCAACAATCAGATGAG[T/C]CTTCTTCAGGACAGGTAATTGACACAGCCAGACGATTTTAAATTAGCTTAGGTTCTATGT |
| NRS266 | R8 | 21347576 | ATCTCTTGTTCGACAGGCTACAATGAATGATGGCTTTATGTTGCAGCAAGGGCAGGATAA[T/G]CAACTTAGCCAAACATTGTTCTCCATGCCATTTACGTCGAGATGCGTTCTTAGATCTTTG |
| NRS267 | R8 | 21348150 | GAGTAAGAGTGCACATCTCGATGACATGAGGTGCTGAGATTCGATGTTTCAGGTATATCG[A/C]GAAGTTGAAACTAGTGGGAGAAACAGTTTGATTTACCACTCAATAACCAAGAAGGGACCT |
| NRS268 | R8 | 27356977 | TTGCAGTAAGACACAAACCTGCTGCATGTGCATTCCATTCTGCAACTGACTAAACTGAGG[A/G]AATTTCTTTCGTAGTGTCTGCATCAAGACCAACGTCGATATATGAGGCAAGAACAAGAAA |
| NRS269 | R8 | 28056202 | GGCAGTATGCTTCCATCCAGAGCTTCCAATTATAATCACAGGGTCCGAAGATGGCACCGT[T/C]CGCATCTGGCATGCAACTACGTACAGGTATTATAATATGAACTCTTTTCTTTGTTAGACA |
| NRS270 | R8 | 29106815 | GTGTTTTTGAGCTGTAGATTGAAAAGCTTAGAACCTGAATTTATAGTTTCCACACCTGAG[A/C]GACTTTTGGAGATTGTTGCCCTCAAAGGAGTTGATATATCGGGGGTTTCGTTACTGGTAT |
| NRS271 | R9 | 114248 | AAAGACTTCCCTGATATCATCTTCGAAACCCAAATCAACCAACCTATCTGCCTCATCCAG[T/C]GTCAGGTACCTATATCAGGAAGGAAAAAGTAAGGCAAACAACATTGTCAACATGTCCAAC |
| NRS272 | R9 | 2182612 | ATGGTAATAAGAAACTTGCGTTAACTTTTCCTACTTACCGAGATTGACACCGGTGTTACC[A/G]ATTTGTGGGTTTGTCATAAGCACAAACTGTCCAGCATAACTAGGATCAGTCAGAATCTCT |
| NRS273 | R9 | 2196243 | ATGGTAATAAGAAACTTGCGTTAACTTTTCCTACTTACCGAGATTGACACCGGTGTTACC[A/G]ATTTGTGGGTTTGTCATAAGCACAAACTGTCCAGCATAACTAGGATCAGTCAGAATCTCT |
| NRS274 | R9 | 4378760 | GCAGCTTAATGAGCCTGTTGAAGTGAAGATAACTGAGTGGAATACTGGTGGGCTTCTCAC[A/G]AGAATTGAGGTAGGAGGAGGCTTCACATGTTTTGTTTTTGAGTTCATACCCCTATTGAAG |
| NRS275 | R9 | 4737350 | TATATTGGGAGCTAATGTCTCGATAATGTTTCAGGTCGTGACATAGGAGTTAATCTTCCA[A/G]TTCTGCTTAATGCCATTGTTAAGGAGCTTCCTTCTGATCAAAGCACAATGCTAAGGATTG |
| NRS276 | R9 | 5770227 | CTATTTGTTACAGAAAGAGCCAGCCCAAGCACTTAACGTGTCTGCCTTACCAATTGATAC[A/C]ATGCTAGAGATGGCTCACCCTCTAATATGTCGTCCTCCCTATGCTTTGTCTTGGCTAACT |
| NRS277 | R9 | 6722869 | GCAAGGGTTGATATTGAGTTGAATCCTTTTTTTAGGACAGGATGATGCATCACAAGTTCT[A/C]CTGTTAGCTACCATATGGATCTTGATTCTCTTGTTTGGTAGCTCAGCATGCGTTTTCCCC |
| NRS278 | R9 | 7216173 | TCTCGATGGGACATGATCATCTTTGTTGCGTATGTCAGTCTCTCTCATTTTACCCCTCTG[T/C]TTAGAGCCCAGATCCTCTTCACGATCCCTTTTTCTAAACCCTTCATCCACACCTCTTCTA |
| NRS279 | R9 | 8342055 | AACTTTCACAGTTGTGATTGTAGGGAACCTTGATCCTAGTATCGCCCTTCCTCTAATTCT[A/C]CAGTATTTGGTAAGTTGGATCACATCACTGGTGATACAAGAAAATAAGTTAGCTCGGACT |
| NRS280 | R9 | 8919822 | CAGGTTATATAAATAGAAAACTCACTTGAGATACTGAAGAGTCATGTTCTTGAGAAGGCT[A/G]GGATGTCTAAGAACAAGGCTTCTCCACTCTTCCTTATCAATCTTTCCATCATGTTTTGTG |
| NRS281 | R9 | 10929368 | AGTAGTTACACTATTCATCTGGTACATCTTTTTGTATTGCTTTCCAAGAGCGTTCTTGGG[T/C]TCGGTAAGCACCTGCAAATAAATAATTTTAGTAATGCGAAACTCACATCAAGTGTCAAAC |
| NRS282 | R9 | 10930499 | TCAAGCACAAGGACAAATTTTTTTAGCTCTAACCTTTTTTGTTATCTTGTCGACTTCATC[A/G]ATGTAAACAATCCCCTGCTGTGCCGCTGCAACGTTATAATCTGCAACCTGGAGAGACAGA |
| NRS283 | R9 | 12559520 | TCTTAGCGTAACAAAGGCGAGAGCAGTACGTGCTTTGTGGTCGAATCTCATCCCAGAGTT[A/G]TCCACGTTGTTCAGCCTTAGAATCTCAGCTAACATTTTTCCAAACTCATCATCACCAACC |
| NRS284 | R9 | 20087006 | AATACCGTTCCCACTGACCCAGAGGTTCAGATCAATGTTACAAAATGGCATCGACTTGAG[T/C]ACTGTCGATGTTAGCTATGTGAGTAGTCGTTCCTGGTAAGTACACATGTTTCATACATGG |
| NRS285 | R9 | 20261141 | ACCTGCGTTGAACTTTGTGGTTGATTTGTAAAGAGGAGTCTTGCAACCTGCACAGCCGTA[T/G]ATTCCTTCTTTGTTGAAATCAACGTATTCTCCGGTTCCTGGATAGCTAAGGCGCGACAAC |
| NRS286 | R9 | 22176885 | CTAAAATTTATTGACTTTTTCCTTTCTTTTTGATTCTTTTTGTCCTTTAGAGACAACTGT[T/G]AGACAAATTGAGGATGCTTTCAAGGAATTCTCATCGAGAGATGATATTGCTATCATCCTC |
| NRS287 | R9 | 22184590 | TGAAAACAAAGAACTCCATTTTCTTCTTAAGAGAAATTAAGTACCTGCTTCTGTGTCAAA[A/G]TTCTCGTCTGCAAGCGCATCCTCCCACTTCTTCCATTGGTGGATCTGCATTAACATAGAA |
| NRS288 | R9 | 24192051 | CTTGTGGCCTTTGAGTTGCATAACTCTTGAAACCTCCTTGACGGAACCATCTTTCTGATA[A/C]ACAATTTCCCAGACCTAGAGAAAAAGCACACGTACAAAGTAGTAAGTCACGCATACATAC |
| NRS289 | R9 | 24448168 | TGGTGCCGCTGGACAGAGATGCATGGCTCTGAGTACAGTGGTCTTTGTTGGCAGTTCAAA[A/G]TCATGGTACCGCGTGCTTACTTTCTTTTCTCCATATTCACATTACACCAAGTCTTTGTAG |
| NRS290 | R9 | 28546320 | GAGAAGTGTTATGGTTTTCATCAACTTCCTCTACACAGTTTTGGTTCTCAATTACTCATC[T/C]GTTGGTTTCATGGTATATATAACCTCTTCCCTCTGTTATTTTTTCGCGCATCTTGTTAAC |
| NRS291 | R9 | 32450518 | TCTTGTAATAGCCTCTGAAACTGTATGTTTTCCGATCCCATAATTCACAGATGACGAAGC[T/C]TCTGAGATACTGTTGGTAAAGAAGAAAAGAGATGAAGTGTTTTTGAAGGACCGTATACCT |
| NRS292 | R9 | 32451474 | TGTACTTTAAACCGGCTTTGGCAAGTTAAAAGGGAGTCATAGTCTCTCATGGCCGCAAAA[A/G]CTGAAGTAATTGTCTTCATATGTTGTGTATATATGTTTCGAAATCTCAGTTCCCAGGAAA |
| NRS293 | R9 | 33015883 | GGTGGTGAGGTAAGTGATTAGGTTGCCAGCGATGCCATAGTAAGCTAGCCTCTCGCAGCA[T/C]TCATTGCCTACACATTAAAAATGTTAGAGAGACAAAAAGAAGGATAAGCCTGCTACATAC |
| NRS294 | R9 | 33017583 | TCCCAAAGGCGGTAGAGAGAAGCGGCAAGGCACTGCACAAGCTCCTCATCGCGTCAGGAG[A/C]CTGCTCGTAGTAGAACTCAAGCTGACCAATGTAGAAGAAAACCTCCGCTGTCCCCATAAG |
| NRS295 | R9 | 33565679 | AACTCTTATTAGACTTCCTTGTCACTTTTAAGTCTCTCAGTTTCTCCTCTTCTTCTTCTT[A/C]TCCTCTTTATCTGCCGAGTTCTTCAGCTGTATAACACAAAGAACTCATCAATGGATGACA |
| NRS296 | R9 | 33571349 | ACTCTTATTAGACTTCCTTGTCACTTTTTAAGTCTCTCAGTTTCTCCTCTTCTTCTTCTT[A/C]TCCTCTTTATCTGCCGAGTTCTTCAGCTGTATAGCACAGAAGACTCATCAATGGATGATA |
| NRS297 | R9 | 34778516 | AAAAAGGTTCTAACTTGTTGCATGGTTTGTGGGGGGTTTTGTAGGTTGCTTCTTTACATC[A/G]TCGGGAGGATTGAGGAGCTACGAGCACCCGAAGAACCAGCTTAAGCCAGTGCCGAGGGTG |
| NRS298 | R9 | 37638549 | GCGAGGAGGAGCCACAACAGCCCTCCCAAGCAAGAACATCCCGCTCAGAGGAGCCTAAAG[T/C]CCGTGAAGACCGGTAAGACAATTCTTTCATCACAAAACCCATGTATTGTCTTTTTCTTAT |

**Supplementary table 5.** The KASP marker set details with upstream and downstream 60bp sequences for 50 SNPs genotyped.

| **SNP Marker Name**  **(Seed purity test)** | **Chr.** | **Genetic distance**  **(position)** | **Probe sequence** |
| --- | --- | --- | --- |
| RsaSPT001 | R1 | 2713383 | AACAAAAAATGGTTTTTAACTGTGGTAATTTTATTATTTTACAGCGCAAGACAAGATTGC[A/G]GATTTTGAGATGAAGCTGATGGATATAGACAGTGAGCATTTGGGGATACCTGATGCTGAG |
| RsaSPT002 | R1 | 14589102 | GGAGAGACCAAATGCAGCGAGTTTTGATAGTTATACCTGAGAAGCAAAGGTAATCTTAAA[A/G]CCAAACACTCGTAACTTTGCTTCTATACGTGGAACCTTCATTAGTTCCATGAAAAACTGA |
| RsaSPT004 | R1 | 19736557 | AAGCGAGAGCTCCAAGCTCTTGAAGCGGCAAATGTACATGCAATTCTGGAAAGCGAACCA[T/C]TAGTTGATGAGGTTATTATTTTTTGTTAATTATTATTTTTAAGACTGTCTTTGCATTTGG |
| RsaSPT005 | R1 | 25861336 | AACAAAAGGTTCCGATCAAAAAATATCAAGTACCTGAGTTAAAGTGATGAAAACATGAAT[A/G]GGCAAAGATGCATCAGAACCAGTAGCCCTCAGCCGGAATTGTGGATTTTGATGCCATGAG |
| RsaSPT006 | R2 | 7798678 | TGTTTCCAGCTCATCAGAATTGGTACTTGAGAACCAGTAACATGTTGCAGGTTGTAAAGG[T/C]CATATCTCTTAGCTGGGTTGTTCAAGAGTGCAATTTGGTGGCGTGGGAGATTGCAGTAAG |
| RsaSPT007 | R2 | 10898597 | ATGTAAACAATATTGAACAATACGATTCAACATACAATCAATCTCAGGCAATCCACTGAT[T/G]AGGAGCTCGAGCTCTTTATCATTAAAAATGGATACGAGCTCACGAGGTATCAACTCATTA |
| RsaSPT009 | R2 | 18530239 | TATCTAGAAAACAGAGGGAAGTGTACTTAACTATTAACATACAAGCAAAAGATTTTACCA[T/C]GAGTAATGTTTTACACATCGGAGAAGTAGTTCGCGAACCGTTATCAAAGCATGAGTGACC |
| RsaSPT010 | R2 | 26179373 | AAGGCAGCTGTTGAAGGGGCTGAGAAGCTATCCGCTGAGCAGTTGGAATTCGCAAAGGAC[A/C]GAATAGTTATGGGCACAGAGAGGAAGACGATGTTCGTATCAGAGGACTCGAAAAAGGTAA |
| RsaSPT011 | R2 | 32418161 | AGAGAAACCAAACGCCTCCCGCCGGAGACCGCAGAACCGGAGATTCCTCGGAGCTGCATT[T/C]GATGAACAGAGCGGACGGTTGGGTGGAGTAAGAGAGGACTGTGAGAAGGAGAGAGAGAAA |
| RsaSPT012 | R2 | 39008624 | ACATATTCGTCTTTTCAAGTGCTTAAAAGTTAAAACAAAACACCTTATTAACTGTTGCTT[T/C]GAGAATTAACAATAGAGAGTGATTTATTCTCTCATTTAGTGACTTAACACTTTCAACCTC |
| RsaSPT013 | R3 | 5841257 | TTTTGACTCATTTCTATAGGAGATTGCATTACTTAGTCGGCTTCAGCATCAGAATATAGT[A/G]AGATATCGTGGCACAGCCAAGGTATGTTCACTTGCAGCTGCCTGAGAATTTTTCTTTGCT |
| RsaSPT014 | R3 | 12187156 | GTTTTAACGCAATCTCGGTGGCCAACACTTCCTCTTGATTTTAAAAGGGATCAATACTAG[A/C]TTGTGGTTGGGGAGAATGAGAAGAGCTATGACAAAAGGTCAAATGTATGTAATTTTGGAA |
| RsaSPT015 | R3 | 16074996 | TTTCATACCTCTAATCTTTAAAACTCTCTTTCTTCTTCTCTCAGATCTGCCAAACTCTGC[A/G]CCAACAACAACAATGGCTTCCGCATCTCCTCCCACCCTCCCAATCTCCAACCCCCAAACC |
| RsaSPT017 | R3 | 20832921 | TGGCTACCTGTAACGTGTGAGGATTTTAATTATTTTTGGTTTTGTGTTGCATATGGCACA[T/C]GCCTGCCTGTCTGCCATAAGGACGGCGCCGCTTTGAACCTGTCCTCGATTTTTGTCTATT |
| RsaSPT018 | R3 | 26159368 | GGACCGGTTCCCATTTGGGTACTTGGCTTCCCTTGTGCTGAAGAAGTACCTCTCCTTTTC[T/G]AAGTTGCCTGCATACAGAAAAAGAGGGCTTCATAAGTCTTAAGCTTGAATTTGAGATTTG |
| RsaSPT019 | R3 | 28952187 | ACCAGCGGTTCCAGCTAAGAAACCTGAGATCATGGATTGCCAAGGGTGCAAGGATTTACC[A/G]TCGCCTTCGTGTTTGTTCCACAAGAGGACGTCAAAGGAGTTTTTGGCAGTGAACATGACA |
| RsaSPT020 | R4 | 3016596 | TCTTCCACCTACTTTAGGTGGCTCCATCTCTGGAGAAACACCGCCGCCACAGTACTGCAA[A/C]ACCGGAGAGTTAAACGAGTTGATTCTCTCCGCCTGCTTCACAGCTCTATGGTCCATCCAA |
| RsaSPT023 | R4 | 12854516 | CACTATAAAGTTAATATCTATGCATGAGACAGGCTGTACCGTCAGTGAGTGCTCCACTTT[T/C]GCGGCTGCATCCAGAAGACGCTGTTCTTCTATAAAAGGCAGTTTTGCTATACCCTTTACA |
| RsaSPT025 | R4 | 17860904 | CGATCCTGTGGCGCTGAAGTTGCTTCAGCGTTTAGTTGCTTTTGACCCAAAGGACCGTCC[A/C]TCTGCTGAAGAGGTAAGTAGAGCAAAAGCTGAAGCTCAGTTTTTCATTCATTTTCATGAT |
| RsaSPT026 | R4 | 25434861 | ACTCTCGTGGATCCAGTGCCATCGCAAGAAGAAGAAACCACCAACGACGTCGTTTGATCC[T/G]TCGCTGTCTTCATCCTTCTCCAACTTGCCTTGCTCTCATCCTTTCTGTAAACCAAGAATT |
| RsaSPT027 | R4 | 32652494 | TTCTGATTTGATTTTCAAACAGCCAAGGCGTGGTTGCTTTGAGATTCGTGAGGAAGGTGG[T/C]GAAACGTTTGTCAGTCTCTTGGTAAGTCTTCCATTTGAGCAACTTTGTTCCCTTTTCAAC |
| RsaSPT032 | R4 | 39217350 | GATACTTTTGGTCTCTTTGCTAAGTGTTACCAATGTAAATCACCGGTTCCAGCTGCCGCC[A/G]CTCATGCTCCGGTCTCTGCTTTACACCACCGCCTTTGAGAGATCTAGCCAGCATATATAA |
| RsaSPT033 | R5 | 3964216 | GAAGAACTCCCTCTGTCTCCTCCTCTCTATAGGCCACAAGTCTCTCCACACCTCAGCCGA[T/G]AGCTGATCTCCTTTAGTAGAACCAAGGTACCTCACTATAGCGTTTGCAACTATCGGCGCA |
| RsaSPT036 | R5 | 12682616 | GTTTAATAAACGCTTATTCTTTCTTTCTTTGTAGGATCATCCTTGGTTCGTAGGTGTGGA[A/G]TGGGGAAAGTTATATCAAATGAAGGCTGCTTTTATTCCCCAAGTCAATGACGAGTTGGAC |
| RsaSPT037 | R5 | 19361198 | TTTCCAGTGTCGGCAATGGAATCACCGAAGCTGATGATGGATTTGAAATTCCGGCATTTG[T/G]TTTCCGAGTTGACGATAGTGAGAAAAAGAGTAGATAAAAAGAAACTAAGGAGCTTCTTCA |
| RsaSPT039 | R5 | 25338280 | GCATTATAATAGCTACCAAGTCTAAAATGGATGCTGATATAATAGGAAAATGGTGGTGAT[T/G]GGGCTGAGCTTGAGGTTCCACTGCCACAATGTTTCATTGATACCATAGGAGAGACAAAGA |
| RsaSPT040 | R5 | 31641574 | GAAGACTCTGCTACTGGTTGGCTTCCTGCTTTCTTCTAAAGCAGCTTCTAGCTCACTTCG[T/C]TTCAACTACGTAGTGAGGAAAAAGCAGGTCAGATTAGATCATACATTGACAAGGAGGTAG |
| RsaSPT041 | R5 | 37737746 | GAGCAGGAGGAGGTGATGAAGAAGGCGCAGAGCCTCGAACCATATGAACGGTGTGATCAG[T/C]CTGCAAACCTAAGGCACAGTTCACAAGAGATTAGAAACAGTAAACGCTCATGAGACAAAT |
| RsaSPT042 | R6 | 7730212 | TCTCAGCTTTTAGACAAGCAGTTGTAGAAATTGAGGATTATCGTCTATTCTGCAGATTTG[T/C]CCTGTATGCAGCGCAGAAGTTTCAACTTCCTCAGCTGGACAAAGCTGAATAGCAAAGGAA |
| RsaSPT043 | R6 | 15210346 | GATTCGTCAACTCTGCTCCAACTCCCGTCAAATCTTCCTCTCTCAACCCAATCTCCTCGA[A/G]CTCCACGCCCCAATCCGCATCTGCGGTAATCTCTCTCTCTTTCTCCCACGATGAAATTGC |
| RsaSPT044 | R6 | 24604213 | AGGAGCGCTTGGTTTTACAGCCAACGTCAAGCCAAGAAAACATTCATCTTGTTTTGTGTT[A/G]AAGTCCCAGAGGTTTAGCTCAAGATTAGGCATCTCCGCTGCTGAATAAAACAAAAAAAAG |
| RsaSPT045 | R6 | 32560826 | TTTTTTTTTAATTTCCTTTTAATTTGTTGTTAACAGAGGGATGACATATCTTCACAATGA[A/G]CCTAATGTTATCATTCACCGAGATCTTAAACCAAGGTAATCACTATTGCATAGCTCTTCG |
| RsaSPT046 | R6 | 38604252 | CCGTCCTAAATCTAAGAGAAACCAGAAGAAAGGCAGGGATTCATCCTCGTCACAGAAGTT[A/G]GATTCGAAAGCAGGAGGCAAGAAAGAGTCCGTTAAGGCGCAAGAAGGTAACAACTTTATC |
| RsaSPT047 | R6 | 44861564 | CTGTTTGATCCGGTTTCCTTGTGTATACAGCTGCAGGATAACATCAGTCTCTTGTATCAG[A/G]CAAGAAATAACATCAGCGCCATTCTGACCGAGTATGTGTCATTGGGTCTTCTTTTCCTTT |
| RsaSPT048 | R6 | 48859791 | ATCACTGCTCGCCTTCACACCGGCTCTTGCCGTCTCCGACAACTCCACCTTCACACCATT[T/C]CCAACGGTGGAGATTGCTGCCACTTGTCCGATCGTCAGAGTCTCTCCTCCTAGATTCACC |
| RsaSPT049 | R7 | 2672570 | CCTTAAAAGTTTTTTTGCTTCACCATGGTTGGTCATGTCATTTAACTGTTTGCAGTGCCC[T/G]GTTCTGATTGCGTGGGGAGAAAAAGATCCATGGGAGCCAATCGAGCTTGGACGAGCTTAC |
| RsaSPT050 | R7 | 7741733 | TTTCCAATGCTTGCTGCTTTATATTCCTGTGTTTTTGGGTTTCCGTCAGGTAAAAGCTGT[T/G]GAGTTAGCAGGTTGTGATTGGATTCATGTTGATGTGATGGATGGTCGTTTTGTTCCCAAC |
| CsaSPT038 | R7 | 12125285 | GAAGAAAGCTCAAAGGGAAAGGGAAGGTGGCCAGGGGGGATCATCTGATGATGATTTCGA[T/C]AGGAGTGGTGGAGCTAGAAGAAGCGCCGAGGACAAGATCAAAGACAATCTGTTTGATGAT |
| CsaSPT039 | R7 | 17943376 | TCCGTGCCACGGTTTGCTGTCCATTGGGTACCAGAATGCAACCGACATTTTGGCTGTTGA[A/G]CCAAGCGTGAACTGAACATCCTGTAGATAAGAATATGTTTTATCTTTAAACACATGTTAT |
| CsaSPT040 | R7 | 26677340 | CAAGGCCTCCGAGATCGGTTTTGACGAGATAATACTTGAATAGAGGCTTGTTGTTGCTGT[A/G]TCTACTTCGACTTGGGTTAACGATGAGGAAACTACAGGAAGACATGCAGACCGAGTTCGA |
| CsaSPT041 | R8 | 4010251 | CTATGCCGAGACGGCAAATGGTACCGAGAATAATCAACTTAGCAAATCAAACGGCACAGA[T/C]CAAGAAGAAGTTGAGGGTGTAGTAGGTAAAAGGCGTTTCCTCGACCTAAACGAGCTTGCT |
| CsaSPT042 | R8 | 11371303 | ACTCCAAACAATTGATCTCTTTCACCCAGGCGTCATCAATCTCGCCTTCCCAGTCGATCA[A/C]AGCCCCGTCTTCTCCCACCACGTTCTTGATGGGATGCGTATCGAAGTACTCCGAAGCTCT |
| CsaSPT043 | R8 | 16763721 | GAACGACGAAAACATCCTCGTACCTCCTCCCACTGAATCAACCATCGACTACATCGCTTC[T/C]GAAAATCTCAAACCGTTTCCAGATCCAGAATCCTCTGTTCAGGTGTCAGATCCTCACCTA |
| CsaSPT044 | R8 | 21348150 | GAGTAAGAGTGCACATCTCGATGACATGAGGTGCTGAGATTCGATGTTTCAGGTATATCG[A/C]GAAGTTGAAACTAGTGGGAGAAACAGTTTGATTTACCACTCAATAACCAAGAAGGGACCT |
| CsaSPT045 | R8 | 29106815 | GTGTTTTTGAGCTGTAGATTGAAAAGCTTAGAACCTGAATTTATAGTTTCCACACCTGAG[A/C]GACTTTTGGAGATTGTTGCCCTCAAAGGAGTTGATATATCGGGGGTTTCGTTACTGGTAT |
| CsaSPT046 | R9 | 5770227 | CTATTTGTTACAGAAAGAGCCAGCCCAAGCACTTAACGTGTCTGCCTTACCAATTGATAC[A/C]ATGCTAGAGATGGCTCACCCTCTAATATGTCGTCCTCCCTATGCTTTGTCTTGGCTAACT |
| CsaSPT047 | R9 | 12559520 | TCTTAGCGTAACAAAGGCGAGAGCAGTACGTGCTTTGTGGTCGAATCTCATCCCAGAGTT[A/G]TCCACGTTGTTCAGCCTTAGAATCTCAGCTAACATTTTTCCAAACTCATCATCACCAACC |
| CsaSPT048 | R9 | 24448168 | TGGTGCCGCTGGACAGAGATGCATGGCTCTGAGTACAGTGGTCTTTGTTGGCAGTTCAAA[A/G]TCATGGTACCGCGTGCTTACTTTCTTTTCTCCATATTCACATTACACCAAGTCTTTGTAG |
| CsaSPT049 | R9 | 32450518 | TCTTGTAATAGCCTCTGAAACTGTATGTTTTCCGATCCCATAATTCACAGATGACGAAGC[T/C]TCTGAGATACTGTTGGTAAAGAAGAAAAGAGATGAAGTGTTTTTGAAGGACCGTATACCT |
| CsaSPT050 | R9 | 37638549 | GCGAGGAGGAGCCACAACAGCCCTCCCAAGCAAGAACATCCCGCTCAGAGGAGCCTAAAG[T/C]CCGTGAAGACCGGTAAGACAATTCTTTCATCACAAAACCCATGTATTGTCTTTTTCTTAT |
