# Supplementary material for: Transcriptome sequencing assisted discovery and computational analysis of novel SNPs associated with flowering in Raphanus sativus in-bred lines for marker-assisted backcross breeding
Source: Hortic Res. 2019 Nov 1;6:120. doi: 10.1038/s41438-019-0200-0 (PMC6823433; doi:10.1038/s41438-019-0200-0)
Supplement: Supplementary file 6 — Supplementary Figure [file 41438_2019_200_MOESM6_ESM.pdf]

Transcriptome sequencing assisted discovery and computational analysis of novel SNPs associated with flowering in *Raphanus sativus* in-bred lines for marker-assisted backcross breeding

Jin-Hee Kim[1 ¥], Abinaya Manivannan[1 ¥], Do-Sun Kim[1], Eun-Su Lee[1], and Hye-Eun Lee[1*]

[1]Vegetable Research Division, National Institute of Horticultural and Herbal Science, Rural Development Administration, Jeonju-55365, Republic of Korea.

*****Correspondence: helee72@korea.kr; Tel.: +82-63-238-6674

¥ These authors equally contributed to this work

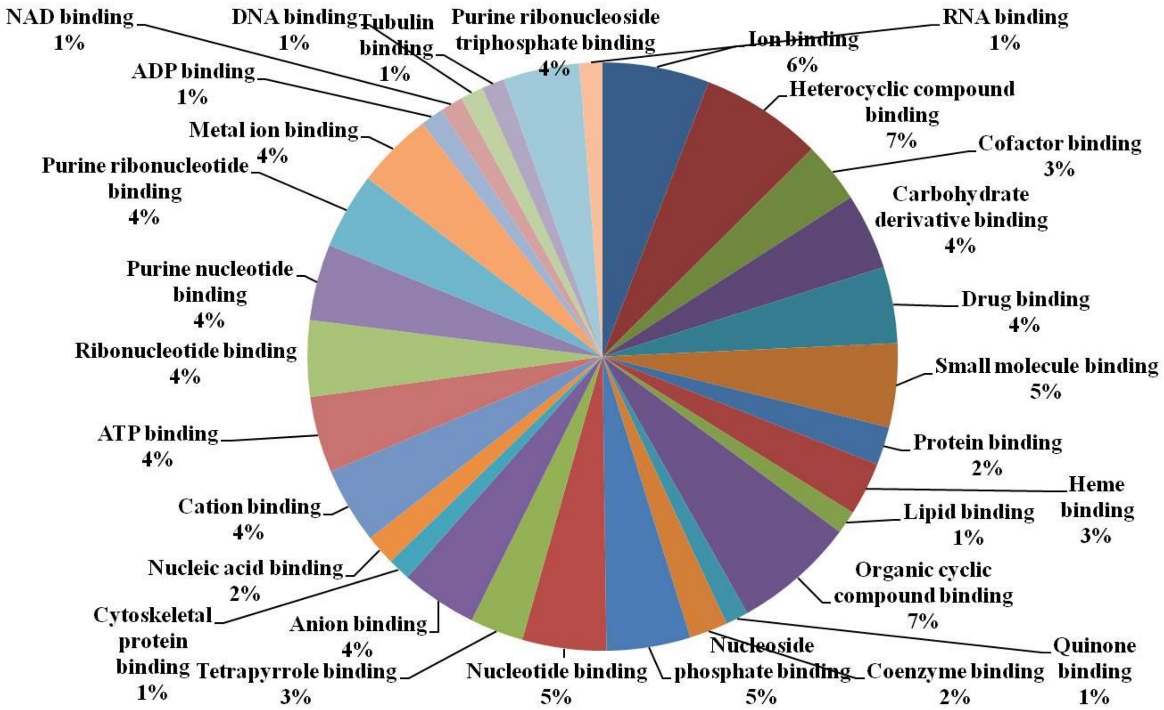

Supplementary Fig: 1. Molecular binding function of SNPs identified in the radish inbred lines further annotated into different levels.
